# Magnetically‐Induced Suppression of Oxidative Stress Prevents Venous Thrombosis

**DOI:** 10.1002/advs.202513299

**Published:** 2025-11-21

**Authors:** Nana Zhang, Yirong An, Shiran Tao, Shenao Qu, Minge Wu, Guantong Fang, Ziqing Chen, Weilun Song, Haoze Leng, Rongqian Wu, Yi Lyu, Xiaoli Liu, Dinghui Dong

**Affiliations:** ^1^ Department of Hepatobiliary Surgery First Affiliated Hospital of Xi'an Jiaotong University Xi'an 710061 China; ^2^ Institute of Regenerative and Reconstructive Medicine Med‐X Institute First Affiliated Hospital of Xi'an Jiaotong University Xi'an 710049 China; ^3^ Key Laboratory for Magnetic Medicine of Shaanxi Province Xi'an 710061 China; ^4^ National Local Joint Engineering Research Center for Precision Surgery & Regenerative Medicine First Affiliated Hospital of Xi'an Jiaotong University Xi'an 710061 China

**Keywords:** oxidative stress, static magnetic fields, vascular endothelium, vascular‐protective effects, venous thrombosis

## Abstract

Venous thrombosis remains a major clinical challenge, with current therapeutic options limited by bleeding risks and procedural invasiveness. Here, it is demonstrated that static magnetic fields (SMFs) provide a non‐invasive and effective strategy for thrombosis prevention by mitigating oxidative damage to the vascular endothelium. In a murine model induced by FeCl_3_, exposure to SMFs significantly reduces thrombus formation, improves survival, and restores venous blood flow without affecting systemic coagulation or fibrinolytic pathways. Mechanistically, SMFs suppressed ROS accumulation and endothelial apoptosis in H_2_O_2_‐challenged vascular cells by elevating intracellular Ca^2+^ levels, which promotes ATP synthesis and suppress NOX4‐mediated oxidative stress. Furthermore, the protective effects of SMF against oxidative stress are largely diminished in the presence of either the calcium channel blocker or the specific NOX4 enzyme inhibitor. These findings reveal a Ca^2+^–ATP–NOX4 signaling axis as a key mediator of the vascular‐protective effects of SMFs, establishing a mechanistic rationale for their antithrombotic action. The consistent efficacy observed across varying SMF intensities underscores their translational potential as a next‐generation thromboprophylactic modality.

## Graphical abstract

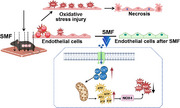

Venous thromboembolism (VTE) remains a leading cause of cardiovascular mortality. This study demonstrates that static magnetic fields (SMF) offer a novel non‐pharmacological intervention against VTE by mitigating endothelial oxidative stress. Mechanistically, SMF elevates intracellular Ca^2+^, boosts ATP production, and suppresses NOX4‐mediated ROS generation, ultimately reducing endothelial injury and thrombus formation, and improving survival in preclinical models.

## 1 Introduction

Venous thrombosis is a prevalent and life‐threatening vascular disorder influenced by diverse risk factors, including genetic predisposition, prolonged immobility, surgery, malignancy, inflammatory conditions, pregnancy, and certain medications.^[^ ^1^ ^]^ It significantly contributes to global morbidity and mortality as a major precursor to myocardial infarction and stroke, and ranks as the second leading cause of death in cancer patients.^[^ ^2^ ^]^ In the United States alone, deep vein thrombosis(DVT) affects up to 600000 individuals annually, with an estimated 244000 deaths attributed to venous thromboembolism.^[^ ^1^, ^3^ ^]^ Current clinical management relies primarily on anticoagulants, supplemented by thrombolytic therapy, mechanical thrombectomy, and physical rehabilitation.^[^ ^4^, ^5^ ^]^ However, anticoagulant and thrombolytic therapies carry substantial bleeding risks, including intracranial hemorrhage, gastrointestinal bleeding, which occasionally result in fatal outcomes.^[^ ^6^, ^7^ ^]^ Although thrombectomy avoids pharmacologic complications, it introduces procedural risks, increases healthcare costs, and causes patient discomfort.^[^ ^8^ ^]^ Given the largely preventable nature of venous thrombosis,^[^ ^9^ ^]^ the urgent need for safer and more effective prophylactic strategies cannot be overstated.

Static magnetic fields (SMF) have garnered attention for their potential to modulate vascular function.^[^ ^10^, ^11^ ^]^ Early studies demonstrated that low‐intensity SMF (1 mT) applied locally could attenuate vasoconstriction and modulate blood pressure responses in rabbits.^[^ ^10^ ^]^ Subsequent clinical studies reported that SMF exposure induced region‐specific alterations in cerebral blood flow in healthy individuals,^[^ ^12^ ^]^ potentially via regulation of sympathetic nervous activity, nitric oxide signaling, baroreflex sensitivity, and calcium homeostasis.^[^ ^13^, ^14^ ^]^ Given that venous thrombosis is fundamentally initiated by endothelial injury, hypoxia, and inflammation, which promotes the expression of adhesion molecules such as vascular cell adhesion molecule‐1, P‐selectin, and E‐selectin.^[^ ^15^, ^16^ ^]^ This cascade facilitates leukocyte and platelet adhesion, activates the tissue factor pathway, and triggers thrombus formation. Magnetic field exposure has been associated with anti‐inflammatory, anti‐edematous, and anti‐oxidative effects in various animal models ^[^ ^17^, ^18^, ^19^ ^]^ raising the possibility that SMF may exert protective effects on the vascular endothelium and prevent thrombogenesis. Despite these observations, few studies have directly explored the antithrombotic potential of SMF. Zhang et al. reported enhanced in vitro clot lysis using magnetic microbubbles under rotating magnetic fields,^[^ ^20^ ^]^ while recent in vivo studies suggest that moderate‐intensity (20–150 mT) SMF exposure may reduce thrombus formation in rodents.^[^ ^21^ ^]^ However, the underlying mechanisms remain poorly understood.

In this study, we investigate whether exposure to SMF can prevent venous thrombosis in a murine model of inferior vena cava thrombosis. We further aim to elucidate the cellular and molecular mechanisms by which SMF exerts its vascular protective effects, with a focus on endothelial oxidative stress. These findings may pave the way for the development of a non‐invasive thromboprophylactic modality with broad translational potential in perioperative and high‐risk clinical settings.

**Scheme 1:**
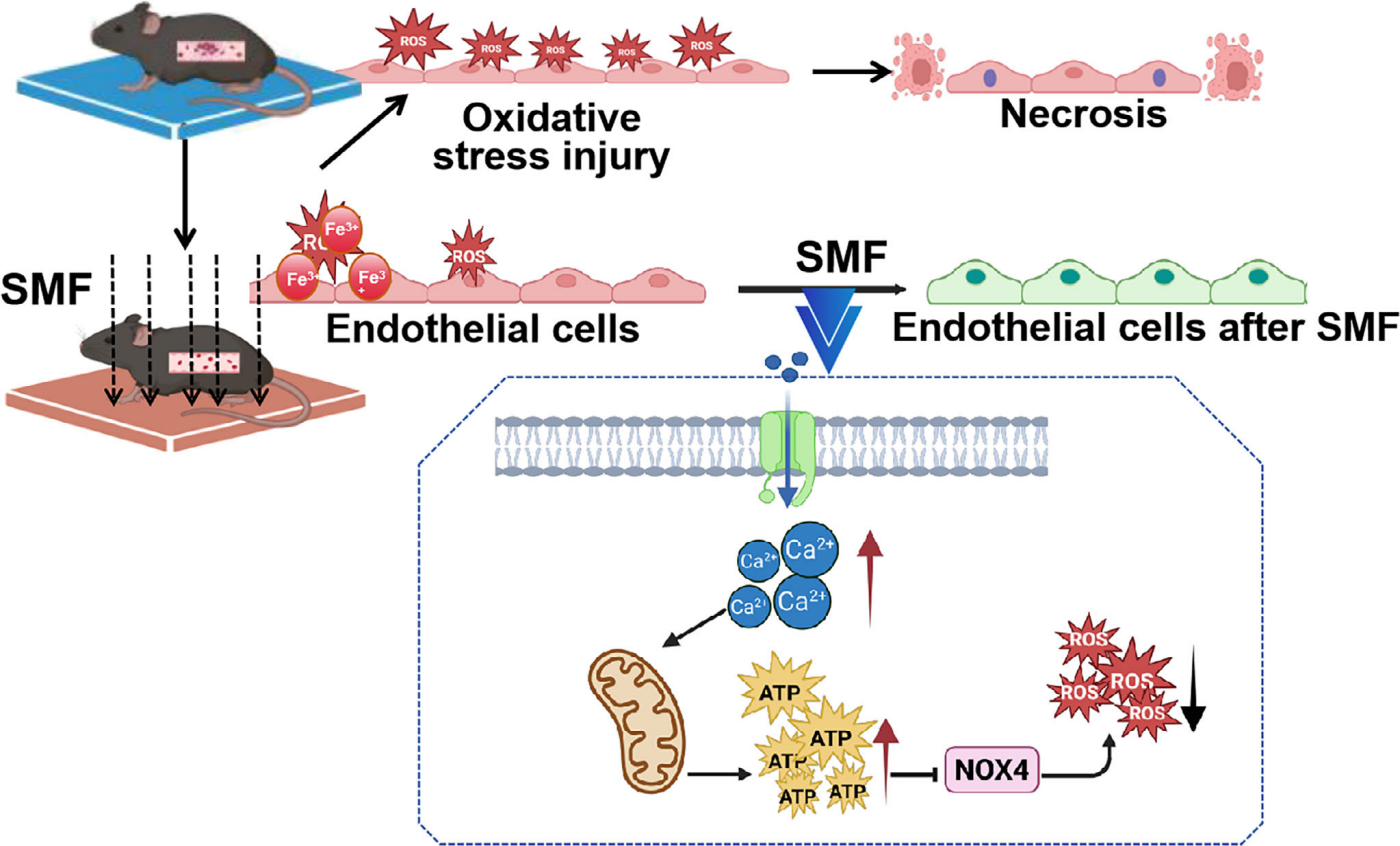
Schematic illustration of the proposed mechanism by which SMF mitigates venous thrombosis via Ca^2+^/ATP‐dependent inhibition of NOX4‐mediated oxidative stress in vascular endothelium.

## 2 Results

**Figure 1:**
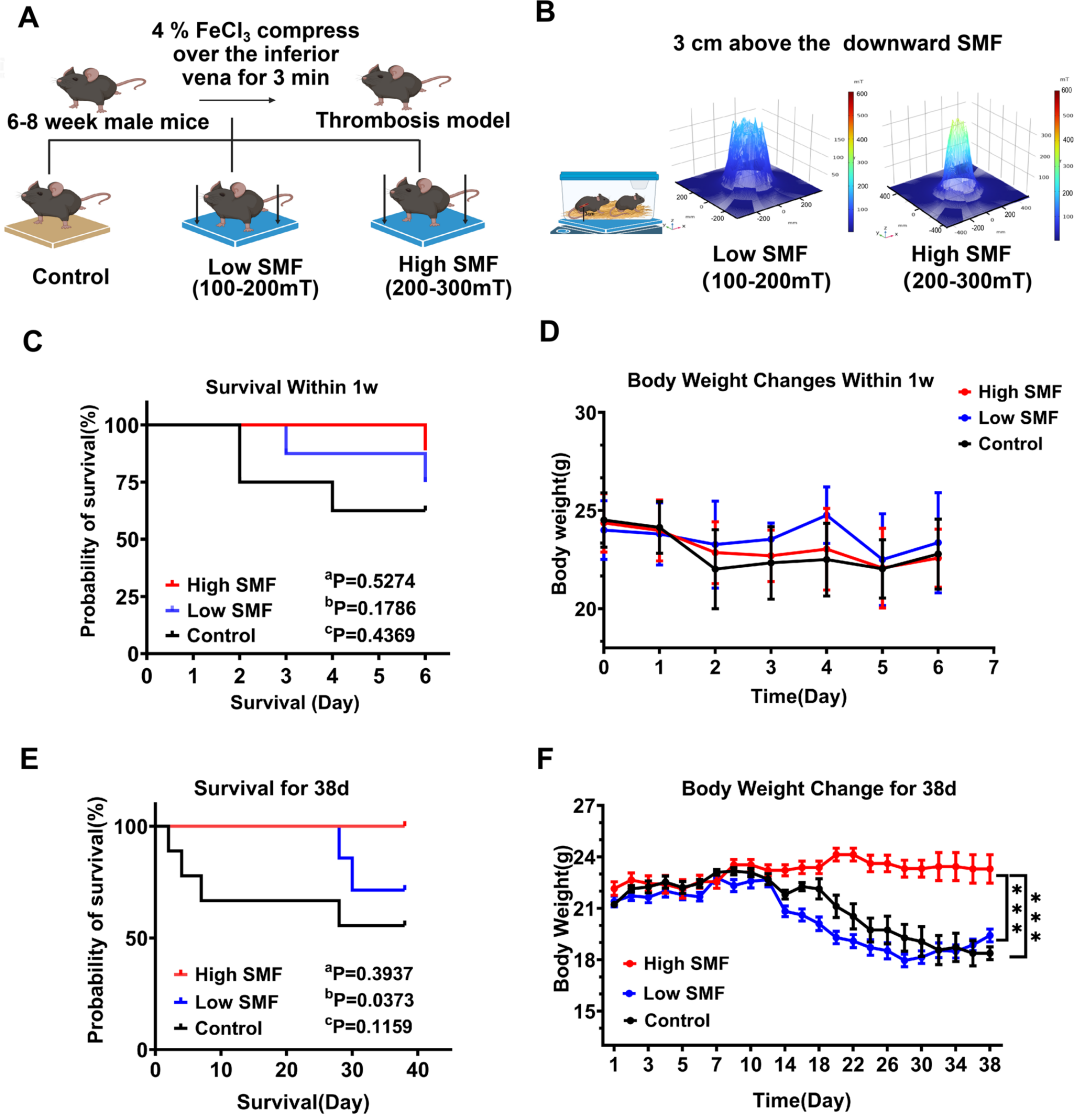
SMF exposure prolongs overall survival and improves the physical condition in mice with FeCl_3_‐induced thrombus. A) Schematic of the experimental workflow. Thrombosis was induced in the inferior vena cava using 4% FeCl_3_ for 3 min. Mice were randomly assigned to low SMF (100–200 mT), high SMF (200–300 mT), or control (no SMF) groups. B) SMF exposure setup. Mice in SMF groups were placed on NdFeB plates generating the designated magnetic fields, while control mice were housed under identical conditions without SMF exposure. C,E) Acute‐phase (one week) and chronic‐phase (38‐day) survival analysis (^a^P. Low group compared versus control group; ^b^P. High group compared versus control group; ^c^P. Low group compared versus High group, n = 7 mice group^−1^, Log‐rank test). D,F) Acute‐phase (one week) and chronic‐phase (38‐day) body mass monitoring (n = 7 mice group^−1^, two‐way ANOVA followed by Tukey's multiple comparisons test. ^***^ *p* < 0.001.

Following induction of thrombosis in the inferior vena cava with 4% FeCl_3_, mice were randomly divided into low SMF (100–200 mT), high SMF (200–300 mT), or control groups immediately upon recovery from anesthesia (**Figure** **1**A). Mice in the SMF groups were placed on an NdFeB magnetic plate generating the designated SMF (Figure 1B), while control animals received no magnetic exposure. Survival was monitored over 38 days, with the 7‐ and 38‐day timepoints demarcating the acute (high‐risk for mortality) and chronic (relevant for long‐term morbidity) phases of the disease, respectively, based on the established clinical course of DVT.^[^ ^22^, ^23^, ^24^ ^]^ The 7‐day acute phase captures the period of active thrombus propagation and significant embolization risk, while the 38‐day chronic phase corresponds to the development of long‐term sequelae such as post‐thrombotic syndrome in murine models. SMF reduced first‐week post‐thrombosis mortality. The high SMF group demonstrated a better survival trend (88.87%) compared to the low SMF (75%) and control (62.5%) groups (Figure 1C), although the intergroup differences were not significant. The body weight was also monitored, but there were no differences in body weight changes among the three groups (Figure 1D). Extended survival analysis revealed the highest early mortality in controls, which gradually declined thereafter.

In contrast, SMF exposure significantly improved long‐term survival, particularly in the high SMF group (Figure 1E). Furthermore, mice in high SMF exhibited significant weight gain compared to both the control and low SMF groups (Figure 1F). At the end of the observation period, all surviving mice underwent necropsy, and no residual thrombosis was detected.

**Figure 2:**
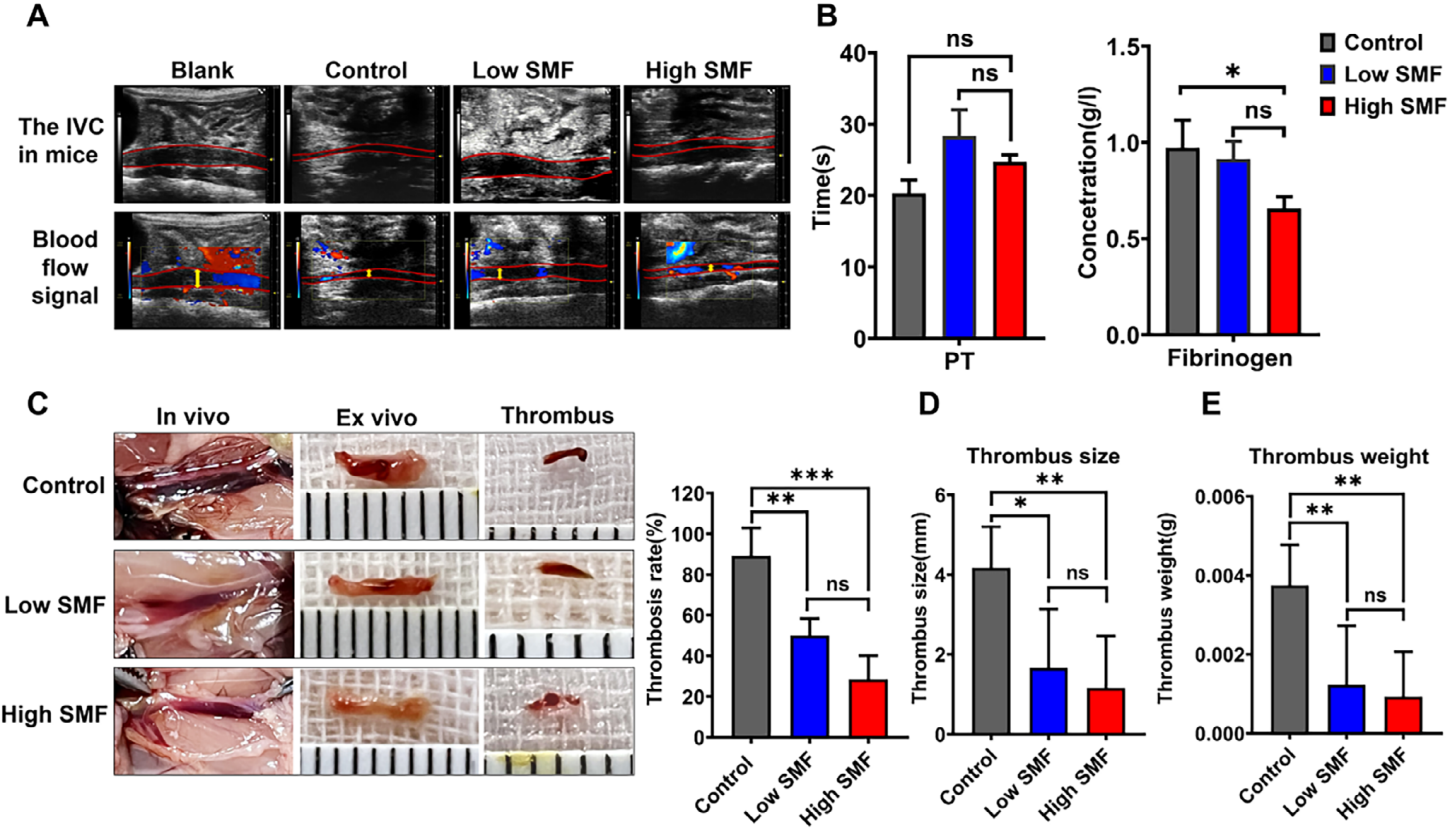
SMF exposure prevents thrombus formation in vivo. A) Representative ultrasound images of IVC (the red line delineates the outline of the IVC) at day 4 post‐thrombosis induction. Thrombus formation with proximal vasodilation is evident in control mice (yellow arrows), while SMF‐treated mice display enhanced blood flow signals and reduced IVC stenosis. B) Analysis of coagulation parameters revealed a significant reduction in FIB levels and a trend toward prolonged PT following SMF treatment (n = 5 per group; one‐way ANOVA followed by SNK test). C) Thrombosis incidence was markedly lower in SMF‐treated mice, occurring in 28% (high SMF) and 50% (low SMF) of animals, compared to 89% in controls (n = 6 per group). D,E) Quantification of thrombus size (D) and wet weight (E) confirmed a significant reduction in thrombus burden in both SMF groups (n = 6 per group; one‐way ANOVA). ^*^ *p *< 0.05, ^**^ *p *< 0.01, ^***^ *p *< 0.001.

Thrombus formation was monitored in vivo using high‐resolution ultrasound beginning on day 4 post‐induction. Due to the limited resolution of ultrasound for detecting small thrombi in mice, thrombus severity was primarily evaluated by assessing blood flow signals in the inferior vena cava (IVC)—the site of thrombus induction. Representative ultrasound images (**Figure** **2**A) depict IVC morphology in each group. In control mice, thrombus formation was evident within the IVC, accompanied by upstream vascular dilation. In contrast, mice exposed to SMFs exhibited more robust blood flow and less pronounced IVC narrowing, suggesting milder thrombosis. Venous blood coagulation parameters detection revealed a trend toward prolonged prothrombin time (PT) and significantly reduced fibrinogen (FIB) levels in SMF‐treated mice (Control vs Low SMF, p = 0.9112; Control vs High SMF, p = 0.0474, Figure 2B), indicating that SMF may attenuate thrombosis by inhibiting the extrinsic coagulation pathway (as reflected by increased PT) and promoting fibrinolysis (as evidenced by decreased FIB). On day 4, the IVC and associated thrombi were harvested for direct evaluation. The incidence of thrombosis was significantly reduced in SMF‐treated groups, occurring in only 28% and 50% of mice in the high and low SMF groups, respectively (Figure 2C). Moreover, SMF exposure significantly reduced both thrombus size (Control vs Low SMF, p = 0.0106; Control vs High SMF, p = 0.0027, Figure 2D) and weight (Control vs Low SMF, p = 0.0080; Control vs High SMF, p = 0.0034, Figure 2E) compared to the control group. Notably, the high SMF group showed greater reductions in both thrombus size and weight compared to the low SMF group. Although these differences were not statistically significant, the high SMF group showed greater reductions in both thrombus size and weight compared to the low SMF group.

**Figure 3:**
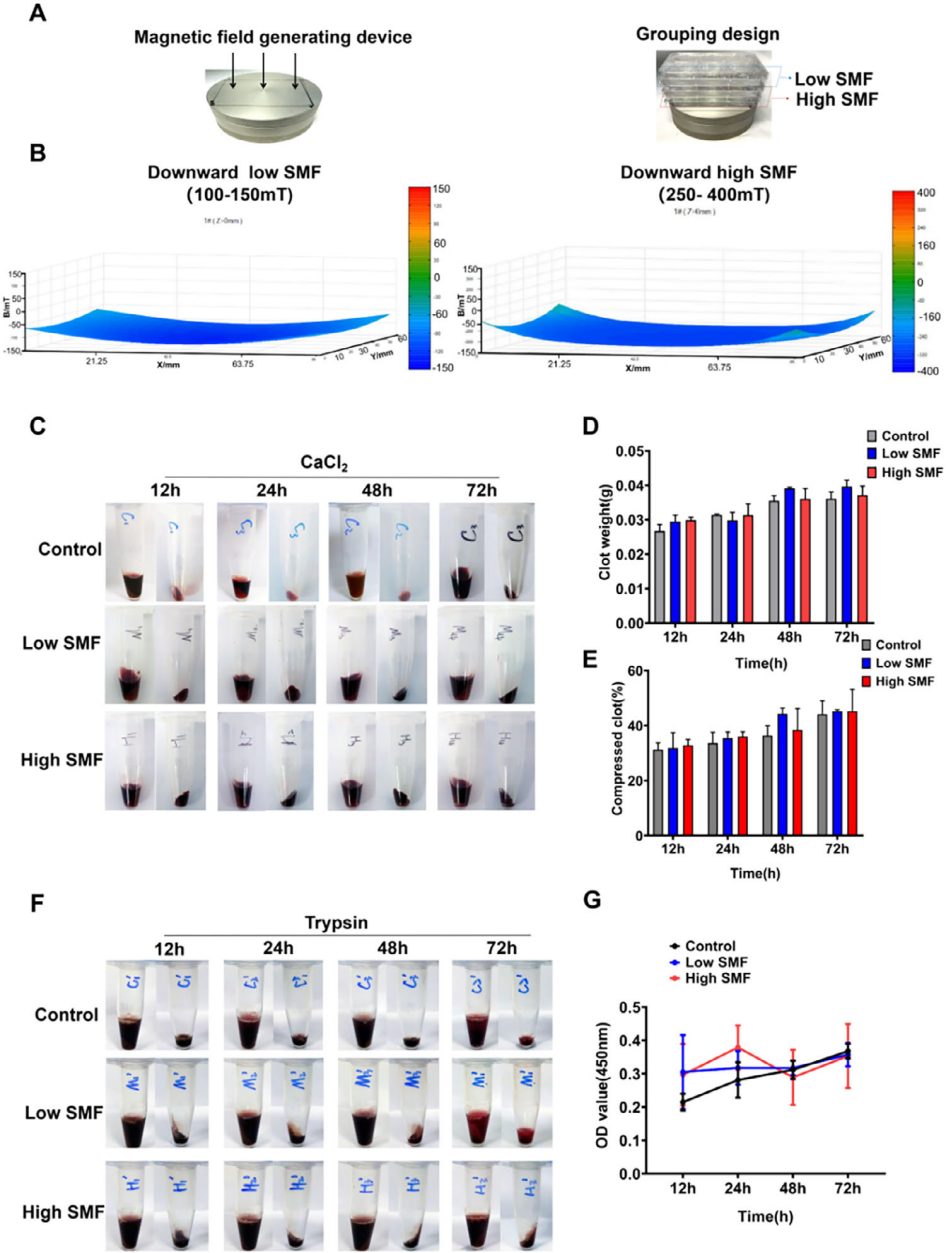
SMFs do not affect blood coagulation or thrombolysis in vitro. A) The magnetic field generating device and grouping design. B) The SMF intervention includes high SMF (250–400 mT), low SMF (100–150 mT), and control (no SMF). C) Gross profile and timeline for WB clot mass and volume measurements. D,E) Quantifying clot mass and volume over time revealed no significant differences between SMF‐exposed and control groups (n = 5 samples group^−1^, two‐way ANOVA analysis with Tukey's multiple comparisons test). F,G) Absorbance measurements indicated no significant variation in hemoglobin release among high SMF, low SMF, and control groups (n = 5 samples group^−1^, two‐way ANOVA analysis with Tukey's multiple comparisons test).

Previous studies^[^ ^25^ ^]^ have demonstrated that hemoglobin contains ferrous ions (Fe^2+^), which impart paramagnetic properties to erythrocytes, allowing them to align directionally under magnetic fields, potentially influencing thrombus architecture. This prompted an investigation into whether SMF might affect blood coagulation or thrombolysis. Whole blood (WB) clots were generated^[^ ^26^ ^]^ across three consecutive 96‐well plates and divided into high SMF (250–400 mT), low SMF (100–150 mT), or control (no SMF) groups (Figure S2A, Supporting Information). SMF‐treated samples were placed on an NdFeB plate generating the appropriate SMF (**Figure** **3**A). Clot mass and volume were measured at 12, 24, 48, and 72 h. No statistically significant differences were observed between the SMF and control groups at any time point (Figure 3C–E). To evaluate potential effects on thrombolysis, WB clots were incubated with 150 µL of trypsin (Figure S2B, Supporting Information), and hemoglobin release was quantified via absorbance measurement. Again, no significant differences were detected among the high SMF, low SMF, and control groups (Figure 3F,G). Collectively, these results indicate that SMF exposure does not significantly influence blood coagulation or fibrinolytic activity under the conditions tested.

**Figure 4:**
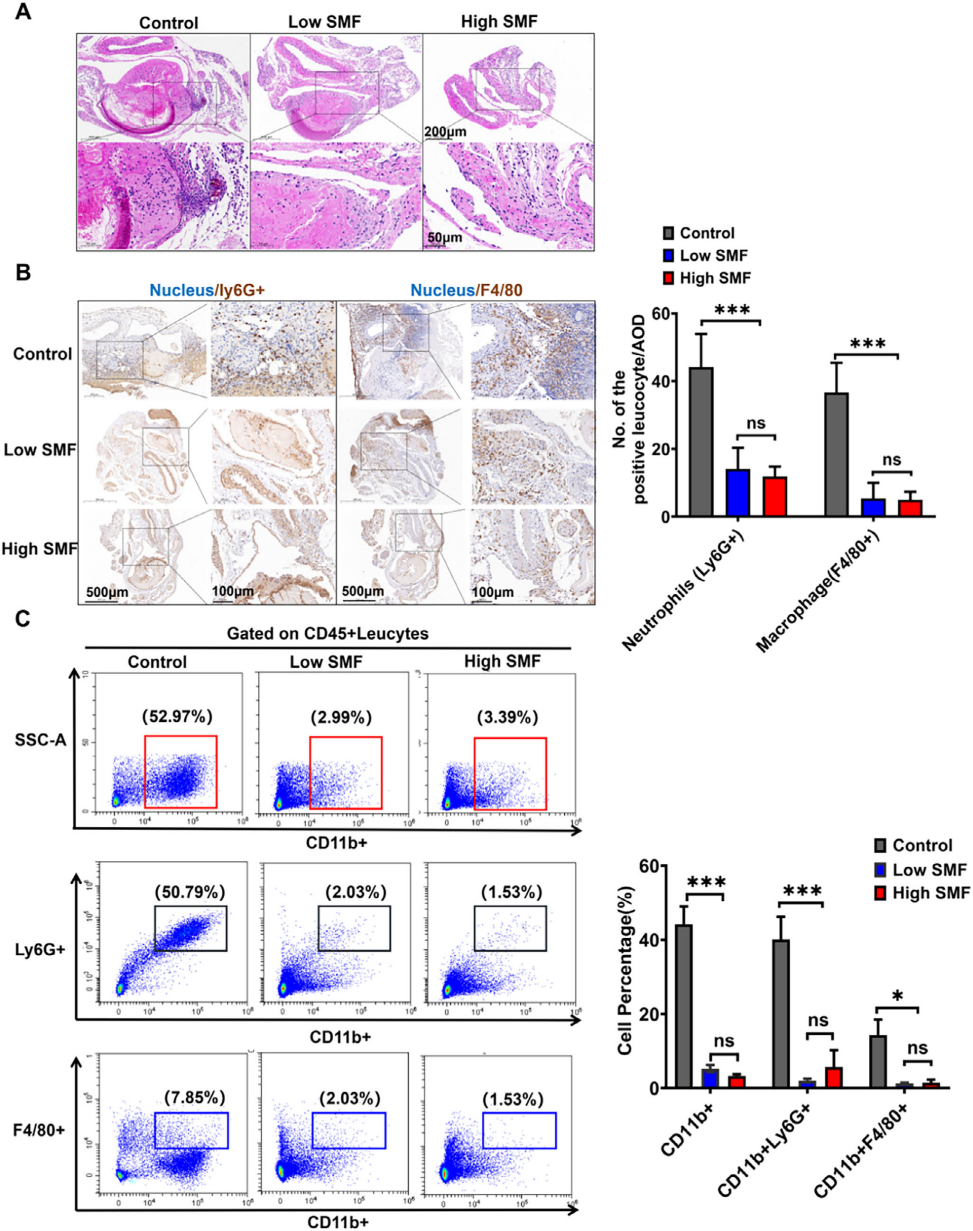
A SMF inhibits inflammatory cell infiltration in the local vessels of the thrombus. A) Histopathological analysis of thrombus‐associated vascular tissue demonstrated reduced inflammatory cell infiltration in the SMF‐treated group compared to the control group. B) Immunohistochemical staining of neutrophils (Ly6G⁺) and macrophages (F4/80⁺) showing significantly decreased infiltration in the SMF group (Ten‐twelve 400 × FOV were randomly recorded and analyzed. Two‐way ANOVA followed by Tukey's multiple comparisons). C) Flow cytometry quantification of leukocyte populations (CD45⁺CD11b⁺), including neutrophils (CD45⁺CD11b⁺Ly6G⁺) and macrophages (CD45⁺CD11b⁺F4/80⁺) in thrombus‐localized vascular tissue (n = 6 mice group^−1^, two‐way ANOVA followed by Tukey's multiple comparisons). ^*^ *p *< 0.05, ^***^ *p *< 0.001.

Histopathological examination showed that SMF exposure markedly reduced inflammatory cell infiltration in thrombus‐associated vessels (**Figure** **4**A). Immunohistochemical staining demonstrated significantly decreased infiltration of neutrophils (Ly6G^+^) and macrophages (F4/80^+^) in SMF‐treated mice compared to controls (Figure 4B). Consistent with these findings, flow cytometry analysis confirmed that SMF treatment significantly reduced the infiltration of total leukocytes (CD45^+^CD11b^+^), including neutrophils (CD45^+^CD11b^+^Ly6G^+^) and macrophages (CD45^+^CD11b^+^F4/80^+^) in vascular tissues at the thrombus site (Figure 4C). These results suggest that SMF inhibits local inflammation in thrombotic lesions.

**Figure 5:**
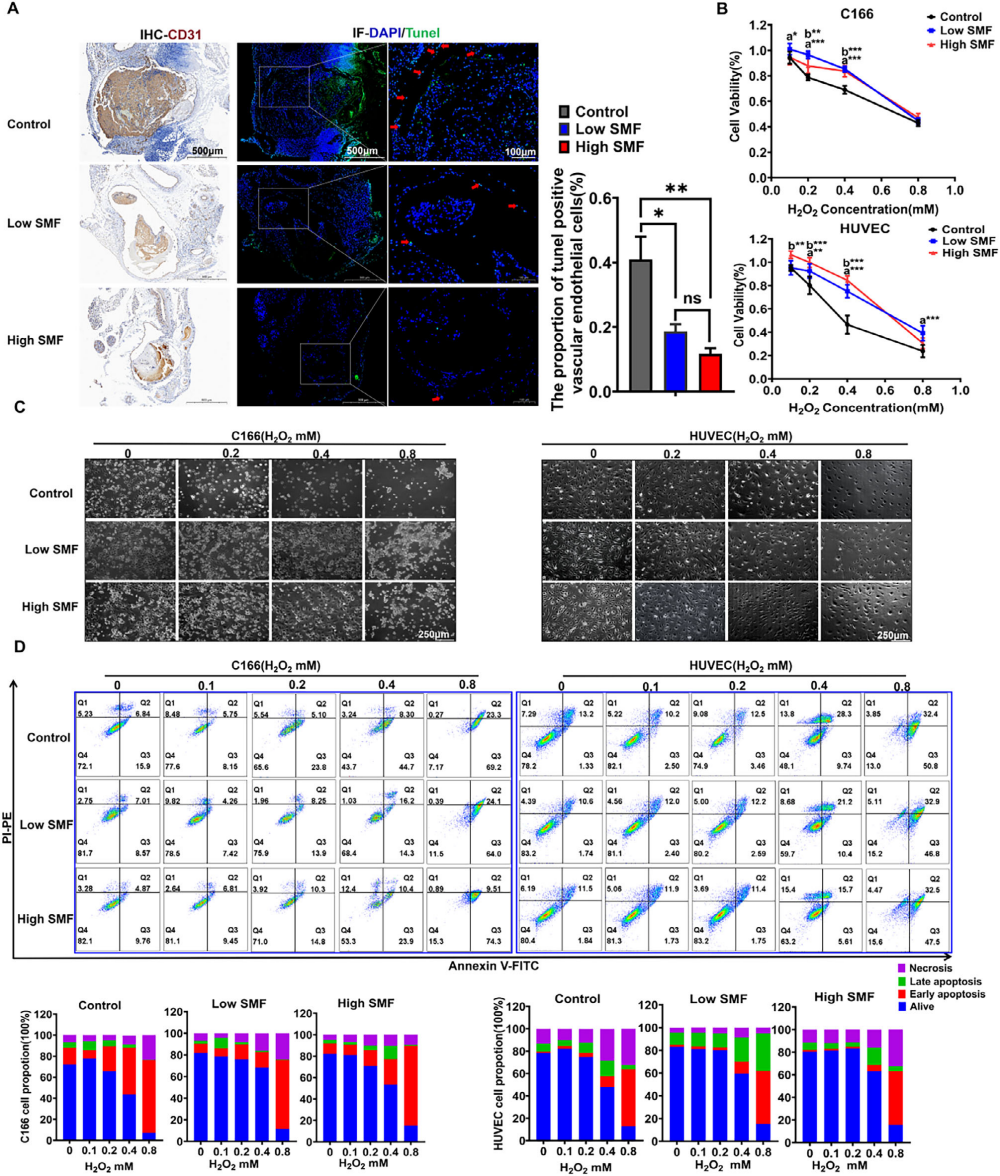
SMF attenuates oxidative stress‐induced vascular endothelial injury. A) In vivo quantification of endothelial cell apoptosis in blood vessels for mice with a thrombus model (CD31‐the specific marker for vascular endothelial cells, with the help of it and histomorphology to locate the location of vascular endothelial cells, and Tunel staining marks cells undergoing apoptosis, positive cells with green color pointed by the red arrow). B) Cell viability of vascular endothelial cells exposed to different concentrations of H_2_O_2_ with the intervention of SMF (a. Low group compared vs control group; b. High group compared vs control group; n = 5, two‐way ANOVA analysis with Tukey's multiple comparisons test). C) Representative cell morphology images under the microscope of C166 and HUVEC endothelial cells under H_2_O_2_‐induced oxidative stress (48 h). D) Flow cytometry analysis for the SMF‐mediated suppression of H_2_O_2_‐triggered apoptosis (n = 3; Flow cytometry for Annexin V‐FITC/PI dual staining). ^*^ *p *< 0.05, ^**^ *p *< 0.01, ^***^ *p *< 0.001.

Given that inflammatory responses are often triggered by cellular injury,^[^ ^27^ ^]^ we next examined whether SMFs could alleviate oxidative damage to vascular endothelial cells. It is well established that FeCl_3_‐induced thrombosis is initiated by Fe^3+^‐mediated oxidative stress, which permeates the vascular wall, disrupts endothelial integrity, and initiates thrombus formation.^[^ ^28^ ^]^ In vivo, SMF exposure significantly reduced the proportion of apoptotic endothelial cells at the thrombotic site (Control vs Low SMF, p = 0.0259; Control vs High SMF, p = 0.0075, **Figure** **5**A). In vitro, a hydrogen peroxide‐induced oxidative injury model was established in both murine (C166) and human (HUVEC) vascular endothelial cells. Cell viability assays (CCK‐8) and microscopic analysis revealed that SMF treatment mitigated hydrogen peroxide‐induced cellular injury (Figure 5B,C), a finding further supported by reduced apoptosis rates in SMF‐exposed cells (Figure 5D).

**Figure 6:**
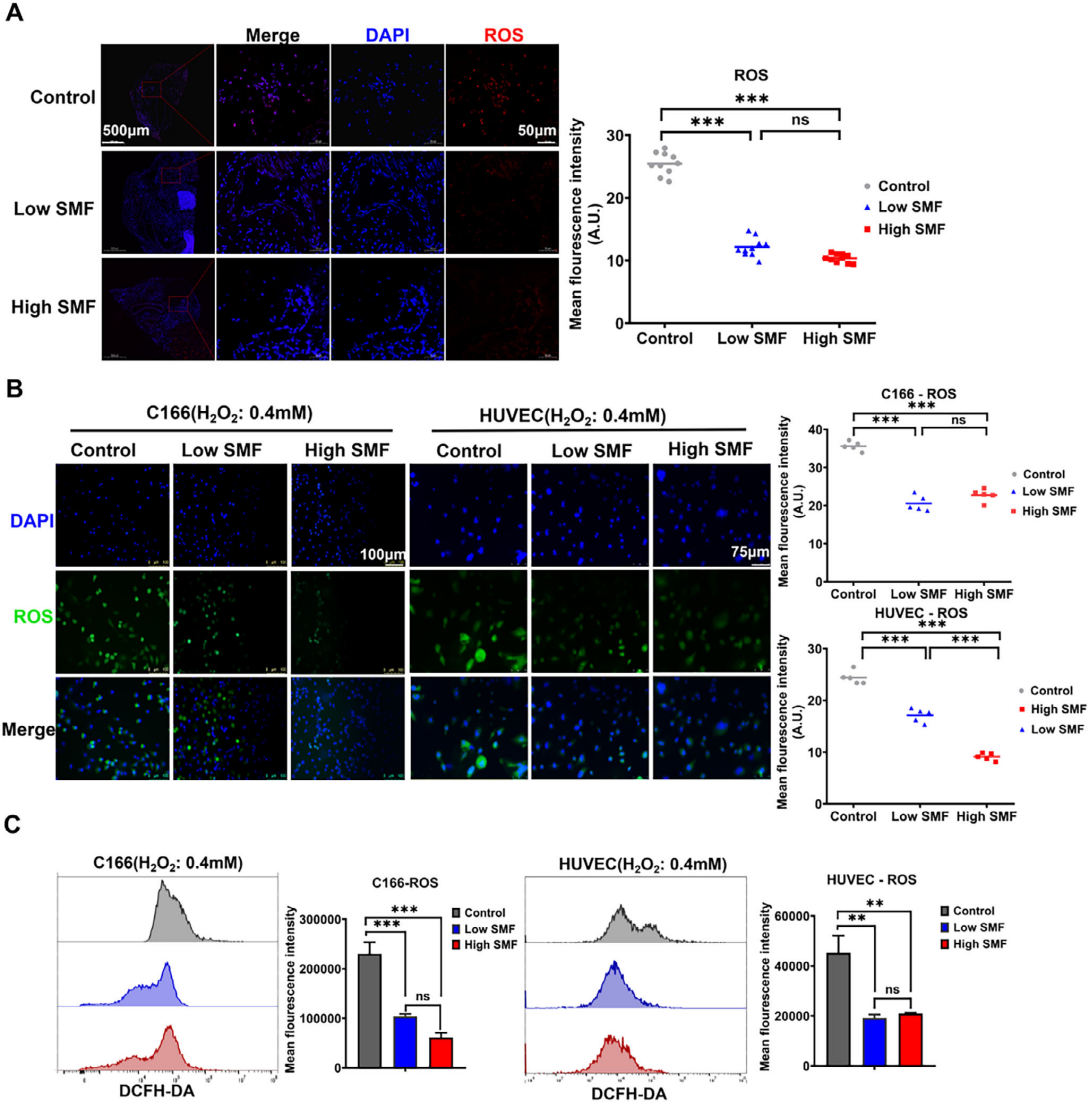
SMF suppresses ROS generation in vascular endothelial cells under oxidative stress. A) In vivo fluorescence staining of ROS levels in FeCl_3_‐injured vascular endothelium(n = 5 mice group^−1^; Ten 400 × FOV were randomly recorded and analyzed. One‐way ANOVA analysis). B) Representative immunofluorescence images of ROS (green) in C166 and HUVEC cells subjected to 0.4 mm H_2_O_2_ (48 h, IF for DCFH‐DA probe staining, five 200 × FOV were randomly recorded and analyzed, one‐way ANOVA analysis). C) Flow cytometry quantification of intracellular ROS levels in C166 and HUVEC cells (n = 3, FCM for DCFH‐DA fluorescence intensity, one‐way ANOVA analysis). ^*^ *p *< 0.05, ^**^ *p *< 0.01, ^***^ *p *< 0.001.

To determine whether SMFs exert their protective effect via suppression of oxidative stress, we assessed reactive oxygen species (ROS) levels in endothelial cells. In vivo, SMF‐treated vessels exhibited markedly lower ROS accumulation in FeCl_3_‐induced thrombotic regions compared to controls (**Figure** **6**A). In vitro, both immunofluorescence imaging and flow cytometry analysis confirmed that SMF significantly attenuated hydrogen peroxide‐induced ROS generation in endothelial cells (Figure 6B,C).

**Figure 7:**
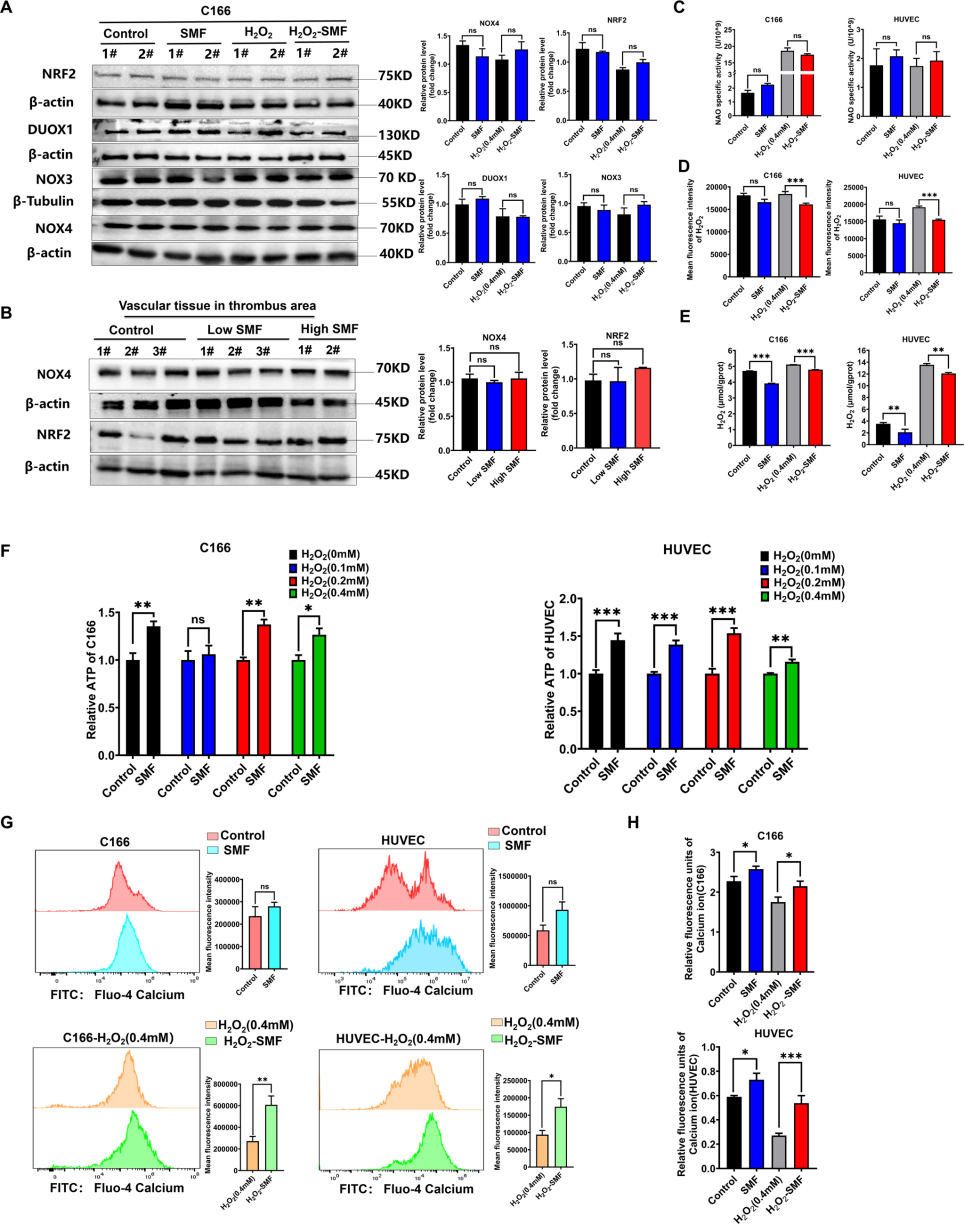
SMF suppresses NOX4‐mediated oxidative stress via the Ca^2+^‐ATP signaling axis. A) Western blot analysis of NOX4, NRF2, DUOX1, and NOX3 in C166 cells treated with SMF (250–400 mT, 48 h) under basal or H_2_O_2_‐induced oxidative stress (0.4 mm, 48 h). (n = 3, β‐actin was used as a loading control; data were analyzed by unpaired *t*‐test). B) Protein expression of NOX4 and NRF2 in vascular tissue at FeCl_3_‐induced thrombotic sites (n = 5 mice group^−1^; one‐way ANOVA). C) Detection for superoxide anion (O_2_−) production by NOX1/2/3/5 isoforms by the colorimetric assay (n = 3, one‐way ANOVA followed by Fisher's LSD test for the indicated pairs). D) Semi‐quantitative evaluation of H_2_O_2_ was measured using the amplex red fluorometric assay (n = 3, one‐way ANOVA followed by Fisher's LSD test for the indicated pairs). E) Quantification of hydrogen peroxide (H_2_O_2_) levels in SMF‐treated cells using a fluorometric assay (Elabscience H_2_O_2_ assay Kit, n = 3, one‐way ANOVA followed by Fisher's LSD test for the indicated pairs). F) ATP quantification in SMF‐treated C166 and HUVEC cells under basal and H_2_O_2_ exposure for 48 h. (n = 3, ATP bioluminescence assay, two‐way ANOVA followed by Tukey's multiple comparisons). G,H) Cytosolic Ca^2+^ levels in SMF‐treated (48 h) C166 and HUVEC cells, measured by Fluo‐4 AM fluorescence. SMF enhances Ca^2+^ influx regardless of H_2_O_2_ exposure (0.4 mm, 48 h) (n = 3, unpaired *t*‐test). ns: not significant, ^*^ *p *< 0.05, ^**^ *p *< 0.01, ^***^ *p *< 0.001.

To elucidate the underlying mechanisms by which SMF attenuates oxidative stress, we investigated key ROS regulatory pathways, focusing on NRF2^[^ ^29^ ^]^ and members of the NOX family.^[^ ^30^ ^]^ Since ROS suppression was similar across SMF intensities, further mechanistic studies employed high SMF (250–400 mT). Protein analysis in C166 cells after 48 h SMF exposure showed no significant changes in NRF2, DUOX1, NOX3, or NOX4 expression under basal or oxidative stress conditions (0.4 mm H_2_O_2_) (**Figure** **7**A), consistent with results of thrombus‐associated vessels showing unaltered NOX4 and NRF2 levels in vivo (Figure 7B). Beyond protein expression levels, the enzymatic activity of NADPH oxidases, particularly that of the highly regulated NOX4 isoform, critically determines the generation of downstream ROS. Owing to the technical limitations associated with direct activity measurement of membrane‐bound NOX enzymes in intact cells, we adopted a well‐validated surrogate method based on the quantification of isoform‐specific ROS products.^[^ ^30^, ^31^ ^]^ Specifically, we measured the generation of superoxide anion (O_2_^−^) as an indicator of NOX1/2/3/5 activity and hydrogen peroxide (H_2_O_2_) as a selective marker of NOX4 activity in endothelial cells, thereby enabling functional inference of respective isoform engagement.^[^ ^32^, ^33^ ^]^ Notably, SMF exposure did not significantly alter O_2_^−^ levels (Figure 7C), whereas a marked reduction in H_2_O_2_ was observed (Figure 7D). It was further confirmed by a quantification method using a fluorometric assay (Figure 7E). These results indicate selective inhibition of NOX4 activity, rather than broad NOX isoform suppression. Since NOX4 is uniquely regulated by ATP via its Walker A motif,^[^ ^34^, ^35^ ^]^ we further assessed ATP levels and found them significantly elevated in endothelial cells under both baseline and oxidative stress conditions following SMF treatment (Figure 7F). To explore the source of elevated ATP, we considered the known influence of SMF on intracellular Ca^2+^ dynamics via voltage‐gated channels,^[^ ^36^ ^]^ and the mild Ca^2+^ elevations are known to activate mitochondrial enzymes and enhance ATP production,^[^ ^37^ ^]^ SMF treatment consistently increased cytosolic Ca^2+^ levels in both cell types, irrespective of H_2_O_2_ exposure (Figure 7G,H). These results support a mechanistic cascade whereby SMF‐triggered Ca^2+^ influx enhances mitochondrial ATP synthesis, leading to allosteric inhibition of NOX4 activity and consequent attenuation of oxidative stress.

**Figure 8:**
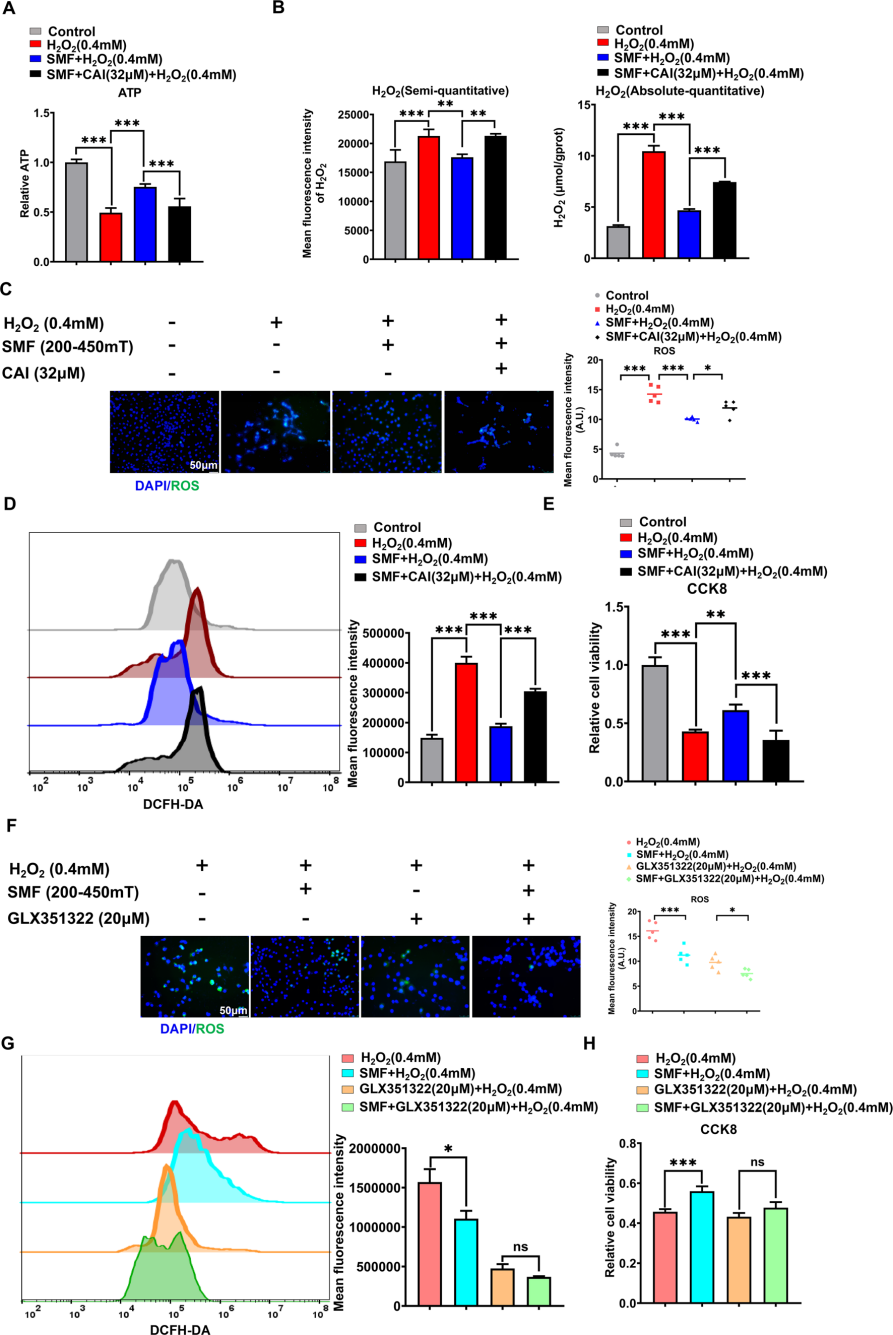
Pharmacological inhibition of the Ca^2+^ and NOX4 enzyme largely diminished the protective effect of SMF, supporting the involvement of the Ca^2+^‐ATP‐NOX4 axis. A) Intracellular ATP levels were quantified in C166 cells treated with or without Calcium channel blocker (Carboxyamidotriazole, CAI) and SMF for 24 h, followed by H_2_O_2_ stimulation for 2 h (n = 53). B) NOX4 enzyme activity was detected by the H_2_O_2_ level change in C166 cells pretreated with CAI for 24 h and SMF, then exposed to basal or H_2_O_2_‐stimulated conditions. NOX4 enzyme activity was assessed by its production of H_2_O_2_ using a fluorometric assay (The absolute‐quantitative: the absolute concentration of H_2_O_2_ can be calculated from a standard curve, Elabscience H_2_O_2_ Assay Kit, and semi‐quantitative methods with Amplex Red fluorescence assay). C,D) Representative immunofluorescence images of ROS (green) C) and Flow cytometry quantification of intracellular ROS levels D) in C166 cells subjected to 24‐h CAI pretreatment and SMF exposure following 0.4 mm H_2_O_2_ exposure (IF for DCFH‐DA probe staining, five 200 ×  FOV were randomly recorded and analyzed, FCM for DCFH‐DA fluorescence intensity, n = 3). E) Cell viability of C166 cells subjected to 0.4 mm H_2_O_2_ with or without 24‐h SMF exposure and CAI pretreatment (n = 5). F,G) Representative immunofluorescence images of ROS (F) and flow cytometry quantification of intracellular ROS levels(G) in C166 cells subjected to 0.4 mm H_2_O_2_ following 24‐h NOX4 enzyme inhibition (GLX351322) and SMF exposure (five 200 ×  FOV were randomly recorded and analyzed, n = 3). H) Cell viability of C166 cells subjected to 0.4 mm H_2_O_2_ with or without 24‐h GLX351322 pretreatment and SMF exposure (n = 5). Data represent the means ± SD from three or five independent experiments. Statistical significance was determined by one‐way ANOVA followed by Fisher's LSD test for the indicated pairs. ns: not significant, ^*^ *p *< 0.05, ^**^ *p *< 0.01, ^***^ *p *< 0.001.

To validate the functional role of the Ca^2+^‐ATP‐NOX4 signaling axis in SMF‐mediated vascular endothelial cells protection, we employed systematic pharmacological inhibition. Calcium channel blockade (Carboxyamidotriazole, CAI, concentration selection as explored in Figure S4A,B, Supporting Information) substantially attenuated SMF‐induced ATP elevation (**Figure** **8**A), and largely abrogated the SMF‐induced NOX4 enzyme activity inhibition (Figure 8B) and also largely abolished its antioxidant effects (Figure 8C) and cytoprotective effect under the (Figure 8D). Similarly, specific NOX4 enzyme inhibition (GLX351322, concentration selection as explored in Figure S4C,D, Supporting Information) hampered the reduction of ROS and cytoprotection by SMF under the H_2_O_2_‐induced cell injury (Figure 8E,F). Specifically, SMF intervention reduced H_2_O_2_‐mediated ROS by 29.54%, whereas the application of a NOX4 inhibitor alone reduced it by 69.69% (Figure 8E). The combination of both treatments resulted in only 76.52% suppression. This non‐additive effect suggests that the SMF exerts its antioxidant effect, at least in part, by inhibiting NOX4. Consequently, the ROS‐suppressive role of the SMF was diminished when Nox4 was pharmacologically blocked, and its cytoprotective effect was ultimately abrogated(Figure 8F). This pharmacological inhibition of the Ca^2+^ and NOX4 enzyme largely diminished the protective effect of SMF, supporting the involvement of the Ca^2+^‐ATP‐NOX4 axis.

## 3 Discussion

Although anticoagulant and thrombolytic therapies remain the mainstay for treating venous thrombosis, their narrow therapeutic windows and high risk of bleeding complications limit broader clinical application. Mechanical thrombectomy, while effective, is invasive and associated with surgical trauma and increased healthcare costs.^[^ ^4^, ^38^ ^]^ These limitations highlight the urgent need for safe, non‐invasive thromboprophylactic strategies. In this study, we demonstrate that SMF can prevent thrombosis by alleviating oxidative damage to the vascular endothelium. Using a FeCl_3_‐induced murine IVC thrombosis model, which mimics ischemia‐reperfusion injury via Fe^3+^‐mediated oxidative stress, we explored the prophylactic potential of SMF in vivo. Our findings reveal that SMF exposure effectively reduced the total mortality rate and slowed the postoperative weight loss rate in thrombotic mice (Figure 1). The SMF intensity (100–400 mT) used in this study was based on comprehensive scientific evidence supporting the use of moderate‐intensity SMF (1 mT–1 T) to elicit measurable biological effects without inducing overt toxicity,^[^ ^24^, ^39^, ^40^ ^]^ which significantly exceeds the Earth's magnetic field yet remains lower than clinical MRI systems. Importantly, many clinical studies have confirmed the safety and physiological relevance of this intensity range,^[^ ^41^, ^42^ ^]^ while in vitro evidence demonstrates enhanced clot dissolution within this spectrum.^[^ ^43^ ^]^

Hemodynamic assessment via ultrasound further confirmed that SMF reduced the thrombosis formation rate to 28% and 50% in high‐ and low‐SMF groups, respectively, while improving IVC blood flow (Figure 2). Given previous reports that ^[^ ^25^ ^]^ hemoglobin‐bound Fe^2+^ confers paramagnetic properties to erythrocytes, it has been hypothesized that SMF could influence thrombus architecture by altering erythrocyte alignment. However, our in vitro assays revealed that neither low (100–150 mT) nor high (250–400 mT) SMF exposure significantly affected clot formation or hemoglobin release during trypsin‐mediated fibrinolysis (Figure 3), suggesting that SMF does not interfere with the coagulation or fibrinolytic cascades directly.

Histopathological analysis indicated that SMF mitigated local inflammation by significantly reducing neutrophil and macrophage infiltration at thrombotic sites (Figure 4). As vascular inflammation is closely linked to tissue injury, we next examined whether SMFs could protect endothelial cells from oxidative stress, a primary pathogenic mechanism of FeCl_3_‐induced thrombosis. Indeed, SMF exposure significantly reduced endothelial apoptosis and ROS accumulation in vivo (Figure 5). Consistent results were obtained in vitro using H_2_O_2_‐induced oxidative injury models in murine C166 and human HUVEC endothelial cells, where SMF treatment preserved cell viability, reduced apoptosis, and suppressed ROS production (Figure 6). These observations align with previous reports showing SMF‐mediated reduction of ROS in neural tissues,^[^ ^44^ ^]^ supporting the notion that SMF preserves vascular integrity through attenuation of oxidative stress.

To investigate the underlying molecular mechanisms, we explored two central oxidative stress regulatory pathways: the Keap1‐NRF2 antioxidant axis and the NOX family. While NRF2 is a key transcriptional regulator of cellular redox balance and has been implicated in SMF responses,^[^ ^29^, ^45^ ^]^ our data showed no significant changes in NRF2, DUOX1, NOX3, or NOX4 expression under basal or oxidative stress conditions in vivo or in vitro (Figure 7A,B). Beyond protein expression levels, the enzymatic activity of NADPH oxidases, particularly that of the highly regulated NOX4 isoform, critically determines the generation of downstream ROS. We adopted a well‐validated surrogate method based on the quantification of isoform‐specific ROS products,^[^ ^30^, ^31^ ^]^ and measured the generation of O_2_^−^ as an indicator of NOX1/2/3/5 activity and H_2_O_2_ as a selective marker of NOX4 activity in endothelial cells, thereby enabling functional inference of respective isoform engagement.^[^ ^32^, ^33^ ^]^ Notably, SMF exposure did not significantly alter O_2_^−^ levels (Figure 7C), whereas a marked reduction in H_2_O_2_ was observed (Figure 7D,E). These results indicate selective inhibition of NOX4 activity, rather than broad NOX isoform suppression. The unique sensitivity of NOX4 to intracellular ATP via its Walker A‐type binding motif^[^ ^34^ ^]^ led us to examine whether SMF modulates ATP levels. Indeed, SMF exposure significantly elevated ATP concentrations in both C166 and HUVEC cells (Figure 7F), suggesting an ATP‐mediated mechanism for NOX4 suppression and downstream ROS reduction. To elucidate the source of this ATP upregulation, we evaluated intracellular Ca^2+^ dynamics. SMF are known to modulate voltage‐gated calcium channels and transient receptor potential channels through physical forces such as Lorentz effects and magnetic torque ^[^ ^35^, ^46^ ^]^ and may prolong calcium signaling by altering calmodulin‐channel interactions.^[^ ^36^ ^]^ Elevated Ca^2+^ levels can promote ATP synthesis by activating mitochondrial dehydrogenases and enzymes of the tricarboxylic acid cycle.^[^ ^47^, ^48^ ^]^ Our findings confirmed increased cytosolic Ca^2+^ in SMF‐exposed endothelial cells under both basal and oxidative stress conditions (Figure 7G,H), suggesting a mechanistic cascade in which SMF enhances Ca^2+^ influx, stimulates ATP production, suppresses NOX4 activity, and thereby mitigates ROS‐driven endothelial injury. The largely abolition of this protective effect through targeted pharmacological inhibition at distinct nodes confirms the causal involvement of the Ca^2+^‐ATP‐NOX4 axis and reveals a novel target for physical intervention (Figure 8).

Despite these promising results, several limitations merit consideration. First, the study focused solely on SMFs, excluding other modalities such as pulsed electromagnetic fields, which have shown therapeutic potential in related settings.^[^ ^49^ ^]^ Inclusion of additional field types could provide a broader understanding of magnetic field biology. Furthermore, the inherent spatial specificity of SMF presents compelling translational promise. While achieving highly localized SMF delivery in small animal models remains technically challenging, future clinical applications could leverage targeted magnetic devices to maximize on‐site therapeutic efficacy while circumventing systemic effects. Second, variability in device architecture, including differences in magnetic field strength, gradient, and spatial distribution, may have influenced the reproducibility of results. The narrow intensity range tested (100–400 mT) may also fall below the threshold required to elicit robust biological effects, as no statistically significant differences were observed between high and low SMF groups despite trends favoring the former. Third, while we identified NOX4 suppression as a key node in the SMF response, further investigation is needed to delineate the precise upstream regulatory network involved in ATP and Ca^2+^ modulation. Additionally, it should be noted that this study utilized male C57BL/6 mice, because our preliminary experiments showed more consistent thrombus formation in males, which is attributable to the recognized protective effects of estrogen in females.^[^ ^50^, ^51^ ^]^ It aligns with clinical epidemiology demonstrating higher DVT incidence in males,^[^ ^52^ ^]^ though we acknowledge this as a limitation regarding the generalizability of findings. Finally, a significant challenge in SMF biology is the lack of standardized instrumentation. Many laboratories rely on custom‐built SMF devices, which vary widely in construction, magnetic intensity, and field geometry. This lack of standardization hampers reproducibility and translational potential across studies.

Taken together, our findings position SMF as a promising non‐invasive strategy for thrombosis prevention through suppression of endothelial oxidative stress. Moreover, existing literature supports that SMFs modulate redox signaling in a spatially selective manner, predominantly influencing tissues directly under field exposure rather than inducing systemic antioxidant effects.^[^ ^53^ ^]^ Establishing standardized experimental platforms and broadening the scope of SMF modalities will be critical for advancing the field. Future studies should also aim to refine the dose‐response characteristics of SMF, characterize long‐term safety, and explore combinatory potential with existing pharmacologic therapies. As understanding deepens, SMF may offer a novel adjunct or alternative in the prevention and management of thrombotic disease.

## 4 Conclusion

Our study identifies SMF as a non‐invasive and biologically active modality capable of preventing venous thrombosis through the protection of vascular endothelial integrity. Using a FeCl_3_‐induced mouse venous thrombosis model, we demonstrate that SMF exposure reduces thrombus burden, improves vascular function, and enhances survival. Mechanistic investigations revealed that SMF mitigates oxidative endothelial injury by suppressing NOX4‐derived ROS production, a process driven by SMF‐induced intracellular ATP accumulation via calcium‐dependent mitochondrial activation. This ATP‐mediated inhibition of NOX4 establishes a previously unrecognized regulatory axis linking bioenergetics and redox homeostasis under magnetic stimulation (Scheme 1). These findings not only shed light on the cellular basis of SMF‐mediated vascular protection but also lay the groundwork for developing SMF‐based strategies to prevent thrombosis and other oxidative stress‐related vascular diseases. Further studies are warranted to define optimal magnetic parameters and evaluate translational potential in clinically relevant human models.

## 5 Experimental Section

### Mice and Inferior Vena Cava Thrombosis Mouse Model

Male C57BL/6 mice (6–8 weeks) were acquired from and housed in the Experimental Animal Center of Xi'an Jiaotong University. All animal procedures were approved by the Institutional Animal Care and Use Committee of Xi'an Jiaotong University (No. XJTULAC2024‐1877). Mice were anesthetized with isoflurane, and the abdomen was opened to expose the peritoneal cavity. The intestines were gently lifted to reveal the inferior vena cava. As shown in Figure S1A (Supporting Information), a 2 × 5 mm piece of filter paper saturated with 4% FeCl_3_ was placed on the exposed inferior vena cava and left in place for 3 min. The filter paper was then removed, and the abdominal cavity was closed.

### SMF Setup

SMFwere generated using two NdFeB plates of different magnetic field strengths in a downward direction. The intensity of SMF was determined based on the value measured at a 3 cm distance from the magnetic plate, corresponding to the approximate distance between the plate and the inferior vena cava in standing mice. For the in vivo SMF exposure, low‐ and high‐intensity SMF groups were exposed to fluxes of 100–200 and 200–300 mT, respectively (**Figure** **1B**). The mice were housed in standard cages and placed at pre‐marked, fixed positions on the magnet surface, corresponding to a region of high spatial uniformity as predetermined by magnetic field simulation. The NdFeB magnet used in this study was a permanent magnetic plate, which generates a static and continuous magnetic field. This unidirectional field was applied vertically in a consistent dorsoventral orientation throughout the experimental period. Control animals were housed in standard cages positioned on non‐magnetic SUS 304 stainless steel plates (relative permeability ≈1.0), ensuring negligible magnetic field exposure while maintaining identical environmental conditions to experimental groups.

For the in vitro *SMF* exposure, cells were exposed to a static, unidirectional downward magnetic field generated between two NdFeB plates, consistent with the in vivo configuration. Field intensities were maintained at 100–150 mT (low) and 250–400 mT (high), and they were centrally verified using a calibrated magnetometer (FE‐2100RD, China). All cultures were positioned within a predefined uniform zone where spatial variation remained below 5%, ensuring consistent exposure. Cells underwent continuous SMF treatment for specified durations under strictly controlled incubation conditions (37 °C, 5% CO_2_), with plates undisturbed throughout exposure.

### Mouse Grouping

Figure 1A illustrates the main parameters of SMF and the grouping of animal experiments. After the establishment of inferior vena cava thrombosis, mice were immediately submitted to the SMF until sacrifice. The mice's weights were recorded every day for short‐term observation and every two days for long‐term observation.

### Assessment of Thrombosis

Based on the preliminary experimental results (Figure S1B, Supporting Information), it was evident that applying 4 % FeCl_3_ for 3 min leads to more prominent and visually identifiable thrombus formation by the third day. Therefore, following three days of housing under magnetic field conditions, thrombus formation in vivo will be assessed using an animal ultrasound imaging instrument (Vevo3100, LAZR‐X, FUJIFILM VisualSonics) with an MX550D linear array transducer. For *ex vivo* assessment of thrombus, the inferior vena cava was isolated, and vessels with attached thrombi were stripped. Thrombus wet weight was determined using a precision analytical balance (Sartorius CPA225D, readability: 0.001 mg). Due to the relatively light weight of the thrombus, the mass of the vessel with the thrombus attached (m1) will be measured first. After detachment of the thrombus, the mass of the vessel tissue (m2) will be measured, with the thrombus mass (m) calculated as m = m1‐m2. The thrombus length was additionally measured by the vernier caliper.

### Whole Blood Clot Preparation

About 10 mL of whole blood (WB) was collected from the inferior vena cava of rats using aseptic blood sampling. The blood was collected into a disposable tube containing 3.2% trisodium citrate as an anticoagulant. WB clots were formed in 96‐well cell culture plates by mixing 100 µL of WB with the same volume of RPMI 1640 and 10 µL of 10% CaCl_2_ solution. All experiments were performed by incubating the WB clots under the influence of SMF or under a condition unaffected by SMF, by placing the cell culture plate on SMF.

### Enzymatic Dissolution of WB Clot by Trypsin

Referring to the previous study, WB clots were generated according to the above protocol (section 2.4) and incubated for 2 h at 37 °C. Following incubation, the supernatant was carefully aspirated, and the resulting pellet was washed twice with 100 µL of phosphate‐buffered saline (PBS) to remove residual plasma. Subsequently, the clots were treated with 150 µL of trypsin for further enzymatic digestion. They were incubated at 37 °C at 12, 24, 48, and 72 h incubation times. Trypsin was neutralized using 150 µL of termination solution (PBS/10% FBS). The supernatant was aspirated and centrifuged at 1300 RPM for 5 min at 4 °C. To ensure hemolysis, the precipitate was resuspended in 150 µL of cold distilled water. The release of hemoglobin was determined using a microplate reader (Thermo Scientific) at 540 nm with the supernatant after diluting the sample 1:5.

### The Evaluation of Volume and Weight of WB Clot

The volume and weight of WB clots were recorded at 0, 24, 48, and 72 h with different treatment conditions. The coagulated clot was gently removed from each well and transferred to a 1.5 mL microcentrifuge tube by microsurgical forceps. In addition, the liquid surrounding each clot was transferred to the same tube. After centrifugation at 16000 × g for 10 min, the volume of liquid was measured, and the weight of the clot was calculated as the difference between the weight of the tube containing the clot and the weight of the tube alone.

### Immunohistochemical Staining

The IHC staining for neutrophils (Ly6G, 1:500, Servicebio Co., Ltd.) and macrophages (F4/80, 1:500, Servicebio Co., Ltd.) was performed after fixation and paraffin embedding, following the manufacturer's instructions. The detailed staining steps are as follows. The slides were dewaxed sequentially with xylene and ethanol. Next, after repairing the antigen under high‐pressure heating, it was washed with a double‐dose washout and PBS, respectively. Next, monoclonal antibodies Ly6G and F4/80 were used as primary antibodies for overnight incubation at 4 °C. HRP Goat Anti‐Mouse/Rabbit IgG (H+L) was chosen as a secondary antibody and incubated at room temperature for 30 min. Color development was achieved by dropwise addition of DAB chromogenic solution, and the staining was counterstained using hematoxylin. Then, gradient dehydration was performed using ethanol and xylene, and the samples were sealed with neutral gum. The inflammatory cell density was evaluated in randomly selected fields under 400 × magnification and quantified using Image J.

### Flow Cytometry for the Inflammatory Cell Infiltration in Vascular

Flow cytometry was used to analyze mononuclear macrophages and neutrophils infiltrating vessels with thrombus in vivo. The vascular tissue wet packed with FeCl_3_ in different treatment groups was harvested, digested with Collagenase D and DNAse I (MilliporeSigma, Germany) for 1 h, and then processed into single‐cell suspensions. The tissue was then filtered, fixed, and stained with fluorescent antibodies [Krome orange anti‐CD45, APC anti‐mouse Ly6G, and PerCP anti‐mouse F4/80 (Biolegend, USA)] for 30 min at 4 °C in the dark. After washing with PBS, cells were analyzed using a flow cytometer (Cytoflex, Beckman, USA). Figure S3 (Supporting Information) details the sequential gating strategy employed to quantify specific immune cell populations.

### Cell Culture and Oxidative Stress Models In Vivo

Mouse‐immortalized vascular endothelial cells (C166) and human umbilical vein endothelial cells (HUVEC‐SV40) were purchased from Procell (Procell Life Science & Technology, China) and incubated at 37 °C in a 5% CO_2_ cell culture chamber. Different concentrations of H_2_O_2_ ranging from 0.1 to 0.8 mm were used to establish the cell oxidative stress model.

### CCK8 Assay

Eight thousand cells were seeded into a 96‐well plate overnight, and the different concentrations of H_2_O_2_ (0, 0.1, 0.2, 0.4, 0.8 mm) were then put into the SMF for 48 h. Cell counting kit‐8 (Abmole, China) was used to detect the in‐vitro proliferative effect of the magnetic field and to identify the degree of injury caused by H2O2 on vascular endothelium, following the manufacturer's instructions.

### ROS Detection In Vitro

Cells were incubated in a 6‐well plate overnight and subjected to SMF for 48 h. To imitate in vivo oxidative stress injury, 0.4 mm H_2_O_2_ was added to the wells before MF treatment. Then, the cells were washed twice with HBSS and incubated with a DCFH‐DA fluorescent probe (Dojindo, Japan) for 30 min. ROS were detected by fluorescence microscopy and flow cytometry.

### Immunofluorescence Staining for ROS Ex Vivo

Cryosections were rewarmed at room temperature, and the autofluorescence quencher was added dropwise for 5 min. ROS staining solution was dropped in the enclosure and incubated at 37 °C for 30 min in a light‐resistant incubator. Nuclei were counterstained with DAPI and washed three times with PBS. Slides were shaken slightly and mounted with an anti‐fluorescence quenching mounting medium. More than 5 Images were captured under a fluorescence microscope and quantified using ImageJ.

### Apoptosis Assay

Annexin V/PI apoptosis kit (Dojindo, Japan) was used for the in vitro apoptosis assay. Briefly, cells were seeded into a 6‐well plate and exposed to different concentrations of H_2_O_2_ (0, 0.1, 0.2, 0.4, 0.8 mm), then subjected to SMF treatment for 48 h. Then, the cells were washed with PBS, digested with trypsin, and incubated with an Annexin V/PI probe following the manufacturer's instructions. Finally, flow cytometry was used for the detection of apoptosis.

### Western Blot

Vascular endothelial cells C166 and HUVEC‐SV40 were treated with 0.4 mm H2O2 for 48 h, with or without SMF treatment. The cells were then washed with cold PBS, lysed with RIPA buffer (Beyotime, China), scraped, and centrifuged at 12 000 RPM at 4 °C for 15 min. A small amount of vascular tissue block with thrombus was placed in a 1.5 mL centrifuge tube, 500 µL of RIPA lysate containing PMSF was added, the tissue was further broken with a homogenizer, and then sonicated with an ultrasound machine, then the centrifuge tube in a 4 °C centrifuge and centrifuged at 12,000 rpm for 25 min. The Bradford protein assay kit (Bio‐Rad, USA) was used to determine the protein concentration in the supernatant. 25 µg of protein was separated by standard sodium dodecyl sulfate‐polyacrylamide gel electrophoresis (SDS‐PAGE) and transferred to a polyvinyl difluoride membrane (Bio‐Rad, USA). The membrane was blocked by 5% skimmed milk for one hour and cultured with primary antibodies specific for NRF2 (Proteintech, 1:2000, Cat#16396‐1‐AP, NOX4 (Immunoway, 1:2000, Cat#YN2975), NOX3 (Immunoway, 1:2000, Cat#YT3175), DUOX1(Immunoway, 1:2000, Cat#YT6883), β‐actin (Immunoway, 1:2000, Cat#YM3028), and β‐Tubulin (Immunoway, 1:2000, Cat#YT4780). After washing three times with PBST solution, membranes were incubated in horseradish peroxidase‐conjugated goat anti‐rabbit or anti‐mouse IgG secondary antibody (Immunoway, 1:20 000, Cat#RS0001) for one hour. The chemiluminescence image was acquired with the ChemiDocTM—touch imaging System (Bio‐Red) and quantified by Image Lab Version 6.1.

### Detection of Intracellular H_2_O_2_

Semi‐quantitative evaluation of H_2_O_2_ was measured using the Amplex Red fluorescent probe (Beyotime, Cat#ST010). Cells were incubated with Amplex Red in the presence of horseradish peroxidase (HRP), and fluorescence was measured at excitation/emission wavelengths of 571/585 nm. Absolute quantification of hydrogen peroxide (H_2_O_2_) levels was determined using the H_2_O_2_ Fluorometric Assay Kit (Elabscience, Cat# E‐BC‐F001, China). The absolute concentration of H_2_O_2_ (µM) was calculated from a standard curve according to the manufacturer's instructions. Briefly, a standard curve was generated with H_2_O_2_ solutions ranging from 0.5 to 10 µm. Samples were mixed with the assay working solution and incubated for 10 min at room temperature in the dark. Fluorescence intensity was measured using a microplate reader at excitation/emission wavelengths of 535/587 nm.

### Detection of Intracellular O_2_^−^

Superoxide anion (O_2_^−^) production by NOX1/2/3/5 isoforms was assessed by measuring NADPH oxidase activity using NADPH Oxidase (NAO) Activity Colorimetric Assay Kit (Elabscience, Cat# E‐BC‐K815‐M). Cells were homogenized in the provided extraction buffer and centrifuged. The supernatant was then incubated with a specific NADPH oxidase inhibitor to define enzyme‐specific activity. Subsequently, the sample was mixed with NADPH and a chromogenic agent to initiate the reaction. After a 10‐min incubation at 37 °C, the absorbance was measured at 450 nm.

### Detection of Intracellular ATP

The Luciferase luminescence assay was used to determine adenosine triphosphate (ATP) in cells using ATP Assay Kit‐Luminescence (Dojindo, Cat#CK18). C166 and HUVEC‐SV40 cells were treated with 0, 0.1, 0.2, and 0.4 mm H_2_O_2_ diluted in DMEM medium and then added to white 96‐well plates, 100 µL of cell suspension was added to each well, and cultured for 48 h in a 5% CO_2_ incubator at 37 °C for 48 h. 100 µL working solution was added to each well, and the wells were shaken for 2 min and then placed at room temperature(≈25 °C) for 10 min, and the luminescence value (RLU) was measured.

### Detection of intracellular Ca^2+^

The C166 and HUVEC‐SV40 cells from different treatment groups were collected and cultured in 6‐ and 96‐well plates. These cells were then subjected to analysis using a fluorescence plate reader and flow cytometry. The specific procedural steps were executed per the operational guidelines provided with the Fluo‐4 Calcium Fluorometric Assay Kit (Elabscience Biotechnology, Cat# E‐BC‐F100).

### Pharmacological Inhibition of the Calcium Channel and Downstream Analysis

Appropriate concentrations of the calcium channel blocker Carboxyamidotriazole (MedChemExpress, Cat# HY‐16126) were determined using CCK8 and the Fluo‐4 Calcium Assay Kit. C166 cells were seeded in 96‐ or 6‐well plates, incubated overnight, and treated with the optimized inhibitor concentration under SMF exposure for 24 h. A concentration of 32 µm was established as optimal, maintaining over 70% viability of endothelial cells while effectively inhibiting calcium influx and reducing intracellular Ca^2+^ levels by more than 50% (Figure S4 A,B, Supporting Information). Cells were subsequently challenged with 0.4 mm H_2_O_2_ for 2 h. Intracellular ATP was quantified using a luminescence‐based ATP Assay Kit. NOX4 activity (detcetion for its production of H_2_O_2_) and cellular ROS levels were evaluated via fluorescence microscopy and flow cytometry. Cell viability under Ca^2+^ channel inhibition, with or without SMF, was measured using CCK8.

### Pharmacological Inhibition of NOX4 Enzyme Activity and Downstream Analysis

The optimal concentration of the NOX4 enzyme activity inhibitor GLX351322 (MedChemExpress, Cat# HY‐100111) was determined as 20 µm using Cell Counting Kit‐8 (Abmole) and a ROS assay kit (Beyotime, Cat# S0033S). This concentration significantly attenuated H_2_O_2_‐induced ROS generation without affecting cell viability (Figure S4C,D, Supporting Information). Cells cultured overnight in 6‐well plates were pretreated with 20 µm NOX4 inhibitor prior to SMF exposure. Following 48 h of SMF treatment, cells were stimulated with 0.4 mm H_2_O_2_ for 2 h. Intracellular ROS levels were assessed by fluorescence microscopy and flow cytometry, and the potential abrogation of SMF‐mediated cytoprotection by NOX4 inhibition was evaluated using CCK8.

### Statistical Analysis

All statistical analyses were performed using GraphPad Prism (v8.0, USA). Data were presented as mean ± SD unless otherwise stated. Unpaired *t*‐test for two‐group comparison to determine differences between groups with normally distributed data. One‐way ANOVA analysis for a three‐group comparison to determine differences between groups. Compare the preselected pairs were determined by one‐way ANOVA followed by Fisher's LSD test. For multi‐group comparison, two‐way ANOVA analysis was used to determine significant differences with Tukey's multiple comparisons test using Graphpad Prism 8.0. All comparisons defined significance as follows: ^*^ *p* < 0.05, ^**^ *p* < 0.01, ^***^ *p* < 0.001.

## Conflict of Interest

The authors declare no conflict of interest.

## Author Contributions

N.Z. and Y.A. contributed equally to the work. N.Z. contributed to the writing of the original draft, methodology, data curation, validation, and funding acquisition. Y.A. contributed to methodology, data curation, and formal analysis. S.T. contributed to methodology and formal analysis. S.Q. contributed to data curation. M.W. contributed to methodology and data analysis. W.S. and G.F. contributed to methodology and data curation. Z.C. contributed to methodology and data curation. H.L. contributed to methodology and data analysis. R.W. contributed to methodology, formal analysis, and funding acquisition. X.L. and Y.L. supervised the study and contributed to writing—review and editing. D.D. contributed to conceptualization, methodology, data curation, writing—original draft, review, and editing.

## Supporting information



Supporting Information

Supplemental File 1

Supplemental File 2

## Data Availability

The data that support the findings of this study are available from the corresponding author upon reasonable request.
