# Supplementary material for: Magnetically‐Induced Suppression of Oxidative Stress Prevents Venous Thrombosis
Source: Adv Sci (Weinh). 2025 Nov 21;13(6):e13299. doi: 10.1002/advs.202513299 (PMC12866686; doi:10.1002/advs.202513299)
Supplement: Supplementary file 1 — Supporting Information [file ADVS-13-e13299-s003.docx]

**Supporting information**

**
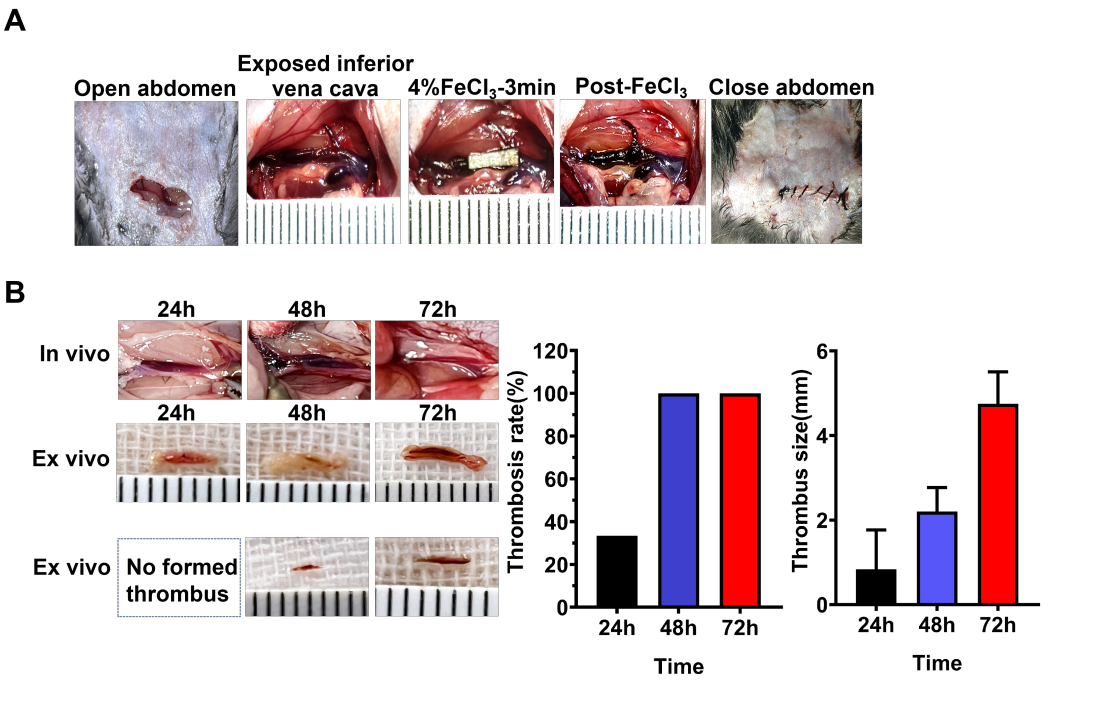
**

**Supplementary Figure S1. (A)** Demonstration of key steps for establishing the inferior vena cava mouse thrombosis model; (**B)** The size of the thrombi changed over time after 4% FeCl_3_ was compressed into the mice's inferior vena cava vessels. Moreover, the macroscopically identifiable venous thrombus could form on the third day.

**
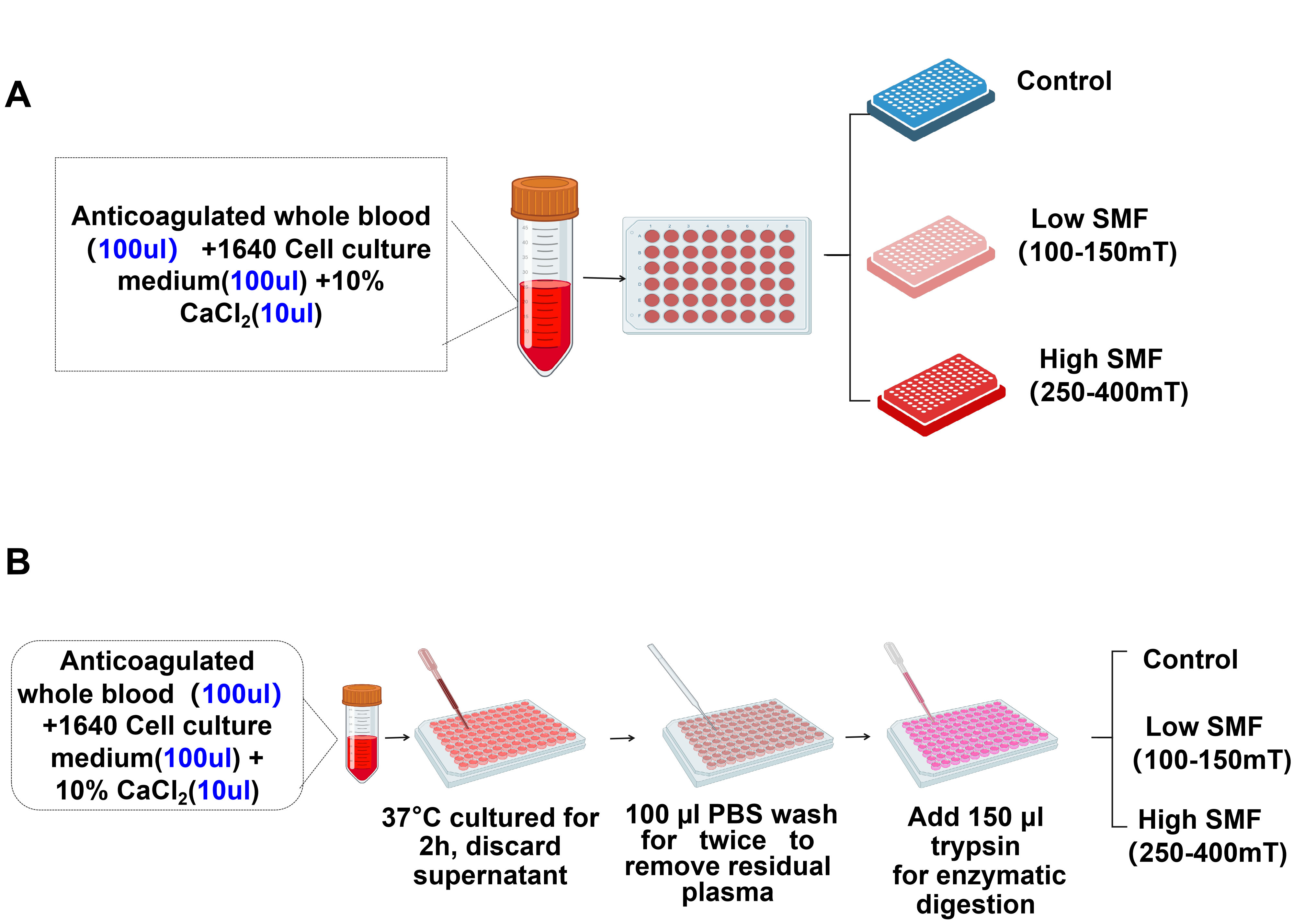
**

**Supplementary Figure S2. (A)** Experimental design schematic: WB clots were prepared in three 96-well plates and divided into three groups; (**B)** Thrombolysis assay design: WB clots were treated with 150μL trypsin to evaluate hemoglobin release.

**
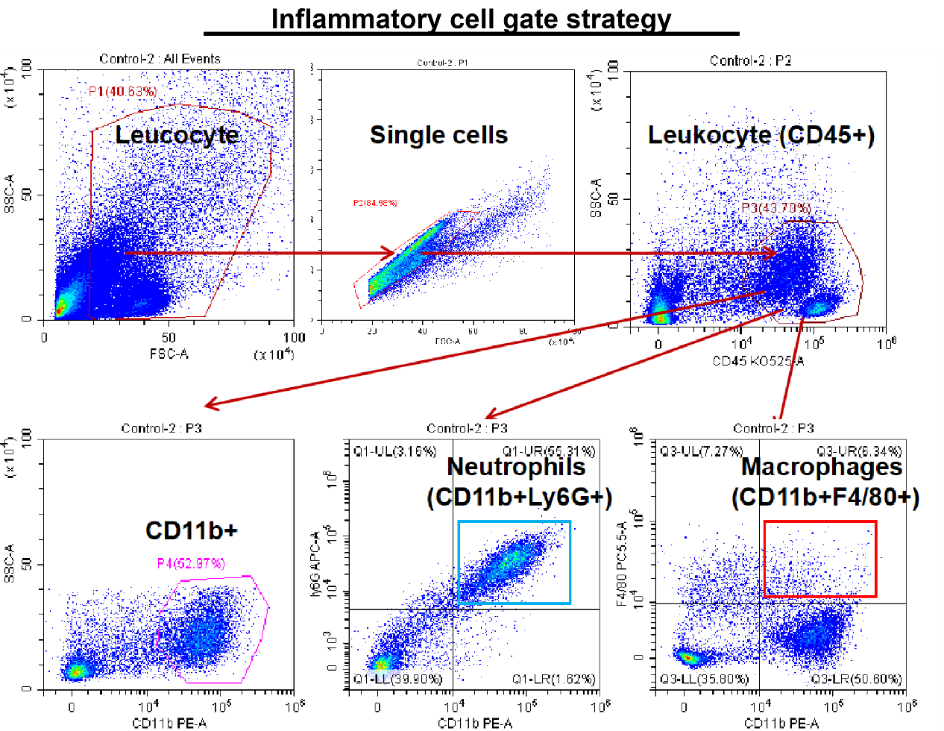
**

**Supplementary Figure S3.** The sequential gating strategy was employed to quantify specific immune cell populations. Within each panel, leukocytes were first identified (Population P1). Doublets and dead cells were subsequently excluded using forward/side scatter characteristics, respectively, yielding a population of live, single cells (Population P2). Panel focused on leukocyte populations. From the CD45^+^CD11b^+^ leukocyte gate within P2, populations of neutrophils (CD45^+^CD11b^+^Ly6G^+^), macrophages (CD45^+^CD11b^+^F4/80^+^), CD11b^+^ and their respective subtypes, were identified. Data are presented as a proportion of CD45^+^ T cells.


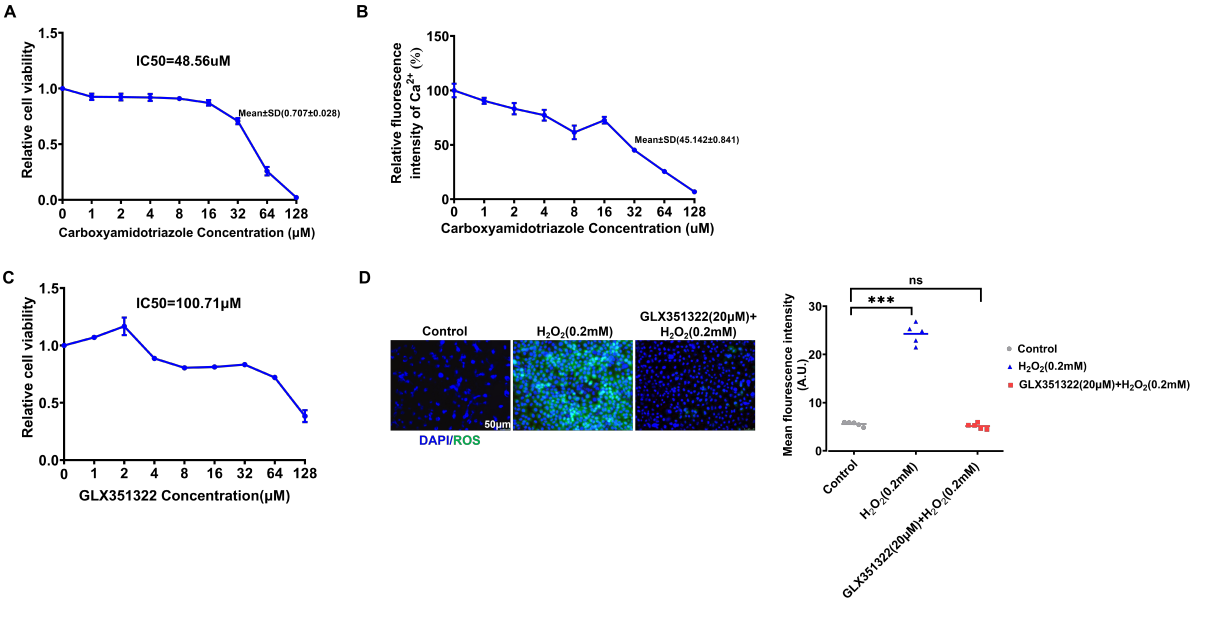


**Supplementary Figure S4.** **Determination of optimal pharmacological inhibitor concentrations.** (A) Cell viability of C166 cells treated with increasing gradient concentrations of Calcium channel blocker (Carboxyamidotriazole, CAI) for 24 h, measured by CCK-8 assay. (B) Intracellular Ca^2+^ levels assessed by relative fluorescence intensity using the Fluo-4 assay. A concentration of 32 µM (indicated by the specific data Mean±SD ) was established as optimal, maintaining over 70% cell viability while inhibiting Ca^2+^ influx by more than 50%. (C) Cell viability of C166 cells treated with increasing concentrations of NOX4 inhibition(GLX351322), measured by CCK-8 assay. (D) Assessment of intracellular ROS levels by fluorescence microscopy (DCFH-DA staining) following H_2_O_2_ challenge. A concentration of 20 µM (indicated by the specific data Mean±SD) was established as optimal, as it significantly attenuated H_2_O_2_ -induced ROS generation without compromising cell viability.
