# Supplementary material for: Magnetically‐Induced Suppression of Oxidative Stress Prevents Venous Thrombosis
Source: Adv Sci (Weinh). 2025 Nov 21;13(6):e13299. doi: 10.1002/advs.202513299 (PMC12866686; doi:10.1002/advs.202513299)

细胞 STR 检测报告

一、样品名称/编号：C166，BNCC317467

二、检测项目：STR 基因型检验

三、检测方法：用天根的基因组抽提试剂盒提取 DNA，采用 18-STR 扩增方案扩增，在 Seqstudio 型遗传分析仪上对 STR 位点和人源位点 TH01、D5S818 进行检测。

四、检验结果：

1. 检验基本情况

多等位基因	匹配细胞系	细胞库	EV 值	匹配说明	人源污染
无	-	ExPASy	-	无匹配	无

多等位基因指三等位及以上基因现象。

本次检测各细胞分型结果良好。

2. 样本描述

该株细胞 DNA 分型在细胞系检索中没有找到匹配的细胞系。本次检测在该细胞系中没有发现多等位基因。

3. 匹配说明

待测细胞系与收录于 ATCC，DSMZ，JCRB 和 ExPASy 数据库的细胞系 STR 数据进行比对，未收录于以上细胞库的细胞系将无法匹配。以下位点中 TH01、D5S818 用于检测该细胞是否存在人源细胞污染。

BNCC
BeNa Culture Collection

附表：C166 细胞的 STR 位点和人源位点的基因分型结果

C166 细胞			
Marker	Allele 1	Allele 2	Allele 3
18-3	17	17	
4-2	19.3	20.3	
6-7	12	15	
19-2	12	12	
1-2	13	13	
7-1	25.2	29	
8-1	13	16	
1-1	10	11	
3-2	13	13	
2-1	9	9	
15-3	22.3	22.3	
6-4	16	16	
13-1			
11-2	16	17	
TH01	—	—	
D5S818	—	—	
17-2	13	17	
12-1	16	16	
5-5	12	14	
X-1	25	25	

附图：ExPASy 数据库比对结果

Export table

Accession	Name	N° Markers	Score	STR 1-1	STR 1-2	STR 2-1	STR 3-2	STR 4-2	STR 5-5	STR 6-4	STR 6-7	STR 7-1	STR 8-1	STR 11-2	STR 12-1	STR 13-1	STR 15-3	STR 17-2	STR 18-3	STR 19-2	STR X-1
NA	Query	NA	NA	10,11	13	9	13	19.3,20.3		16	12,15	25.2,29	13,16				22.3		17	12	
CVCL_0120	3T3-Swiss albino	12	64.71%	10,15	13	9	14	19.3	13,15	15.3	12,15	25.2,29	15,16	15	19	15	20.3	12,15	17,21	12	26,27
CVCL_VR92	MEC1	12	61.11%	11	13	9	12,13,15	19.3	13,14	18.3	12,15	29	16,17	15,18	17,18	15	20.3,21.3	13	17,20	12,14	25

分型方案及位点分布

	方案 1	方案 2	方案 3	方案 4
1	18-3	1-2	2-1	TH01
2	4-2	7-1	15-3	D5S818
3	6-7	8-1	6-4	17-2
4	19-2	1-1	13-1	12-1
5		3-2	11-2	5-5
6				X-1

检测人：张葡萄

审核人：殷世腾

签发日期：2022.09.16

河南省工业微生物菌种工程技术研究中心

Henan Engineering Research Center of Industrial Microbiology

网址：www.bncc.org.cn 电话：400-6699-833

9.15mouse

Sample File	Sample Name	Panel	SQI	OS	SQ
A1_A1_Sample_20220915_093828.fsa	A1	MRMouse 18 Kits_Panels_v2.0			

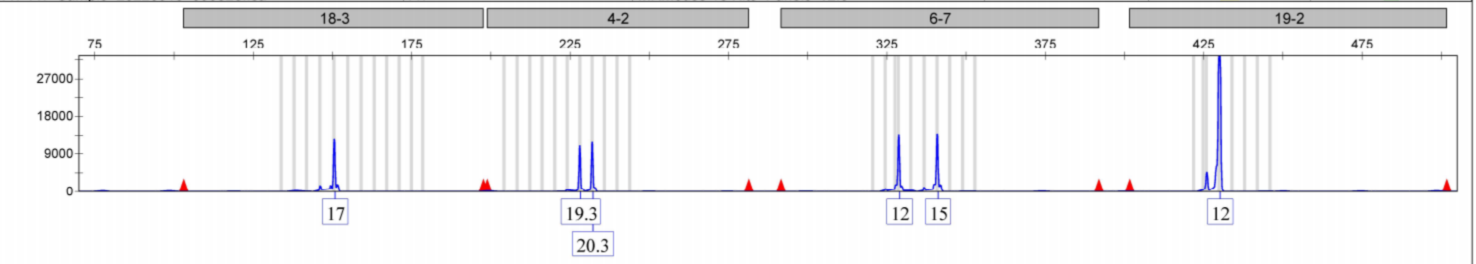

Sample File	Sample Name	Panel	SQI	OS	SQ
A1_A1_Sample_20220915_093828.fsa	A1	MRMouse 18 Kits_Panels_v2.0			

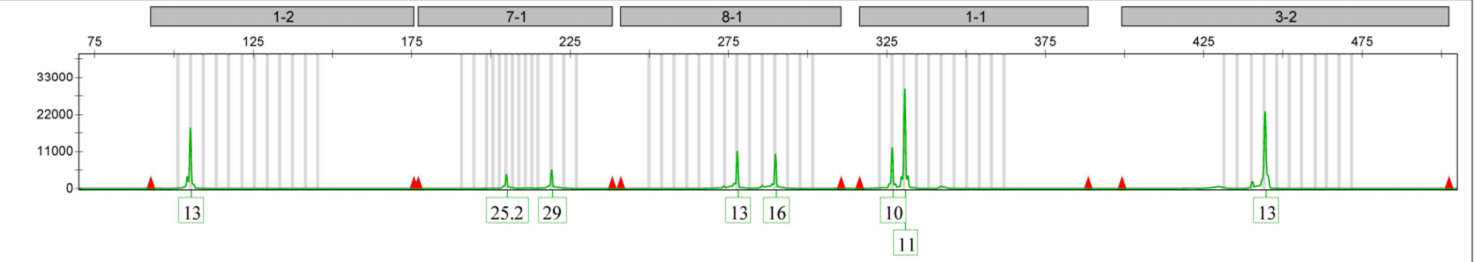

Sample File	Sample Name	Panel	SQI	OS	SQ
A1_A1_Sample_20220915_093828.fsa	A1	MRMouse 18 Kits_Panels_v2.0			

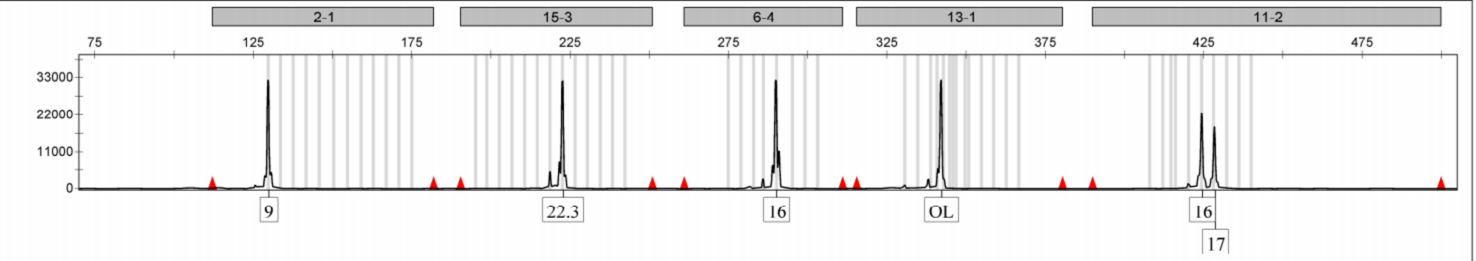

Sample File	Sample Name	Panel	SQI	OS	SQ
A1_A1_Sample_20220915_093828.fsa	A1	MRMouse 18 Kits_Panels_v2.0			

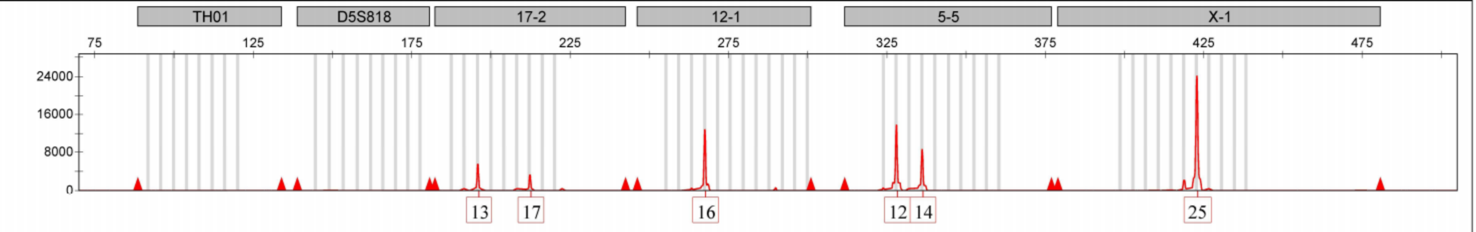

Supplement: Supplementary file 2 — Supplemental File 1 [file ADVS-13-e13299-s001.pdf]
