# Supplementary material for: Magnetically‐Induced Suppression of Oxidative Stress Prevents Venous Thrombosis
Source: Adv Sci (Weinh). 2025 Nov 21;13(6):e13299. doi: 10.1002/advs.202513299 (PMC12866686; doi:10.1002/advs.202513299)
Supplement: Supplementary file 3 — Supplemental File 2 [file ADVS-13-e13299-s002.pdf]

NO: JDBG2403156

镜像绮点(上海)细胞技术有限公司

免 疫 荧 光 鉴 定 报 告

检品名称：HUVEC

检验单位：镜像绮点

检验类型：免疫荧光鉴定

报告日期：2024 年 3 月 1 日

样品编号：人脐静脉内皮细胞 HUVEC

检测项目：免疫荧光鉴定

试验所需仪器设备及试剂

（1）仪器

仪器名称	规格型号	厂家
生物安全柜	BSC-1500ⅡA2-X	济南鑫贝西生物技术有限公司
CO_2细胞培养箱	BC-J160S	上海博迅实业有限公司
荧光倒置显微镜	DS-Ri2	Nikon
高速冷冻离心机	Multifuge X1R	Thermo Fisher
电热恒温鼓风干燥箱	DHG-9123A	上海精宏实验设备有限公司

（2）试剂耗材

试剂名称	规格/货号	厂家
T25 细胞培养瓶	430639	Coming
血球计数板	Neubauer improved	Marienfeld
24 孔板专用细胞爬片	YA0350	Solarbio
细胞培养孔板	WHB-24	上海卧宏生物科技有限公司
胎牛血清	1414426	Gibico
内皮细胞完全培养基	PriMed-iCell-002	镜像绮点（上海）细胞技术有限公司
0.25%胰蛋白酶（含 0.02%EDTA）	1734858	Gibico
多聚甲醛（PFA）	P1110	Solarbio
DAPI	C0060	Solarbio
Triton X-100	T8200	Solarbio
山羊血清	S9070	Solarbio
CD31	28083-1-AP	proteintech

Goat anti-Rabbit lgG（H+L）<br>Cross-Adsorbed Secondary antibody,<br>Alexa Fluor 594	SA00006-3	proteintech
Fluoromount-G 荧光封片剂	0100-01	SourthernBiotech

实验步骤

（1）细胞爬片

取 3 片玻璃片于 24 孔板中，每孔加入培养基 1mL，加入细胞 0.02million 个/孔。置培养箱 2h 或过夜。

（2）固定

细胞爬片后，吸出培养基，用 PBS 洗 1 遍，加入 4% PFA 于 4℃固定 30min。用 PBS 洗 3×5min/次。也可最后一次不吸出 PBS，放 4℃过夜。

（3）破膜封闭

将玻片除去水分，置于培养皿支撑物上，

玻璃片封闭液配置：0.5% Trition X-100 与 PBS 1:1 混合，再加 10% 血清，

取 50uL 破膜封闭液滴于防水膜上，将玻片上有细胞的一面盖上 2h。

（4）一抗孵育

一抗配制：抗体与 PBS 1:100（200）稀释

破膜封闭后，取 50uL 一抗于防水膜上（湿盒中），将玻片（有细胞的一面）盖上置于 4℃（最多可放置一周）

（5）二抗孵育

室温避光孵育二抗（二抗:PBS=1:500）2h 后，PBS 洗 3×5min/次，染 DAPI（DAPI:PBS=1:1000）5min，PBS 洗 3×5min/次。

（6）包埋

玻片上各滴 1 滴 Fluoromount-G，将有细胞的一面盖上。

检验结果：

（一）　细胞免疫荧光鉴定照片：

CD31：

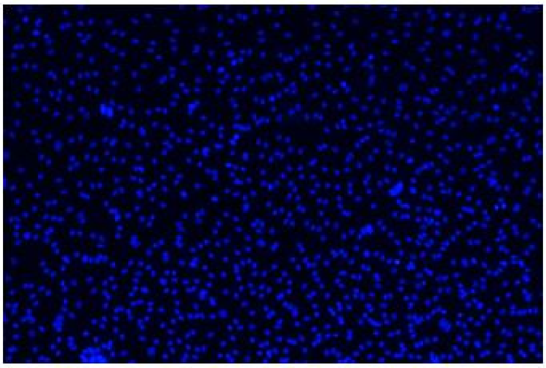

100X-DAPI

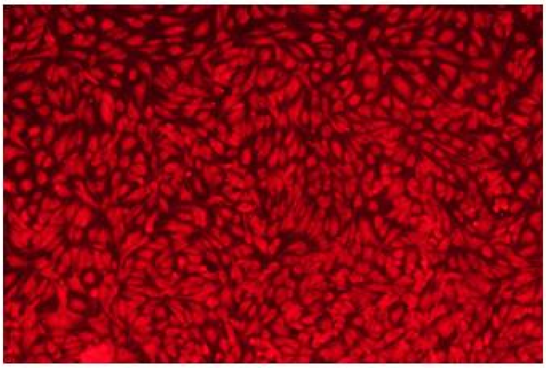

100X-Fluorescence

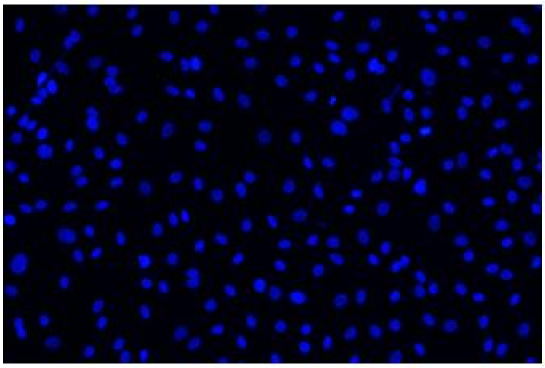

200X-DAPI

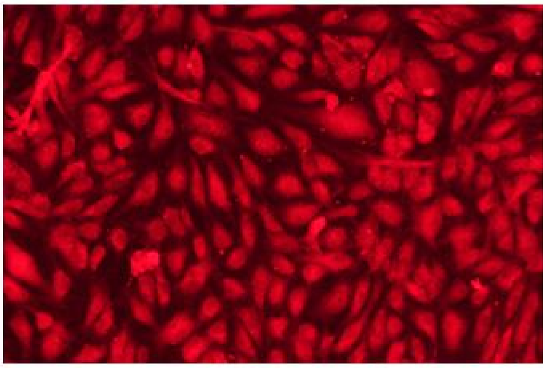

200X-Fluorescence

（二）　检验基本情况：

经免疫荧光鉴定，该细胞纯度达到 90%以上

说　　明

1. 本报告只对送检的来样负责。
2. 检验报告上的检验结果和检验单位名称，未经同意不得用于广告、评优及商业宣传。
3. 对本报告有异议，请于收到报告之日起十五日内以书面方式提出，逾期不予受理。
4. 对纸质检验报告涂改、增删，或未加盖检验单位印章的复印件均无效。

检测单位：镜像绮点（上海）细胞技术有限公司
联系地址：上海市奉贤区茂园路 260 号 56 号楼 7 楼
邮政编码：200231
联系电话：400-021-2021

镜像绮点（上海）细胞技术有限公司 报告专用章

签发日期：2024 年 3 月 1 日

镜像绮点（上海）细胞技术有限公司
注册地址：上海市奉贤区金海公路 6055 号 11 幢 5 层
办公地址：上海市奉贤区茂园路 260 号 56 幢 7 层
电话：400-021-2021
网址：www.icellbioscience.com
